# Developmental Stage, Solid Food Introduction, and Suckling Cessation Differentially Influence the Comaturation of the Gut Microbiota and Intestinal Epithelium in Rabbits

**DOI:** 10.1093/jn/nxab411

**Published:** 2021-12-07

**Authors:** Martin Beaumont, Eloïse Mussard, Céline Barilly, Corinne Lencina, Laure Gress, Louise Painteaux, Béatrice Gabinaud, Laurent Cauquil, Patrick Aymard, Cécile Canlet, Charlotte Paës, Christelle Knudsen, Sylvie Combes

**Affiliations:** GenPhySE, Université de Toulouse, INRAE, ENVT, F-31326, Castanet-Tolosan, France; GenPhySE, Université de Toulouse, INRAE, ENVT, F-31326, Castanet-Tolosan, France; GenPhySE, Université de Toulouse, INRAE, ENVT, F-31326, Castanet-Tolosan, France; GenPhySE, Université de Toulouse, INRAE, ENVT, F-31326, Castanet-Tolosan, France; GenPhySE, Université de Toulouse, INRAE, ENVT, F-31326, Castanet-Tolosan, France; GenPhySE, Université de Toulouse, INRAE, ENVT, F-31326, Castanet-Tolosan, France; GenPhySE, Université de Toulouse, INRAE, ENVT, F-31326, Castanet-Tolosan, France; GenPhySE, Université de Toulouse, INRAE, ENVT, F-31326, Castanet-Tolosan, France; GenPhySE, Université de Toulouse, INRAE, ENVT, F-31326, Castanet-Tolosan, France; Toxalim (Research Centre in Food Toxicology), Université de Toulouse, INRAE, ENVT, INP-Purpan, UPS, Toulouse, France; GenPhySE, Université de Toulouse, INRAE, ENVT, F-31326, Castanet-Tolosan, France; GenPhySE, Université de Toulouse, INRAE, ENVT, F-31326, Castanet-Tolosan, France; GenPhySE, Université de Toulouse, INRAE, ENVT, F-31326, Castanet-Tolosan, France

**Keywords:** milk, weaning, bacteria, metabolites, development

## Abstract

**Background:**

In mammals, the establishment around weaning of a symbiotic relationship between the gut microbiota and its host determines long-term health.

**Objectives:**

The aim of this study was to identify the factors driving the comaturation of the gut microbiota and intestinal epithelium at the suckling-to-weaning transition. We hypothesized that the developmental stage, solid food ingestion, and suckling cessation contribute to this process.

**Methods:**

From birth to day 18, Hyplus rabbits were exclusively suckling. From day 18 to day 25, rabbits were *1*) exclusively suckling; *2*) suckling and ingesting solid food; or *3*) exclusively ingesting solid food. The microbiota (16S amplicon sequencing), metabolome (nuclear magnetic resonance), and epithelial gene expression (high-throughput qPCR) were analyzed in the cecum at days 18 and 25.

**Results:**

The microbiota structure and metabolic activity were modified with age when rabbits remained exclusively suckling. The epithelial gene expression of nutrient transporters, proliferation markers, and innate immune factors were also regulated with age (e.g., 1.5-fold decrease of *TLR5*). Solid food ingestion by suckling rabbits had a major effect on the gut microbiota by increasing its α diversity, remodeling its structure (e.g., 6.3-fold increase of Ruminococcaceae), and metabolic activity (e.g., 4.6-fold increase of butyrate). Solid food introduction also regulated the gene expression of nutrient transporters, differentiation markers, and innate immune factors in the epithelium (e.g., 3-fold increase of nitric oxide synthase). Suckling cessation had no effect on the microbiota, while it regulated the expression of genes involved in epithelial differentiation and immunoglobulin transport (e.g., 2.5-increase of the polymeric immunoglobulin receptor).

**Conclusions:**

In rabbits, the maturation of the microbiota at the suckling-to-weaning transition is driven by the introduction of solid food and, to a lesser extent, by the developmental stage. In contrast, the maturation of the intestinal epithelium at the suckling-to-weaning transition is under the influence of the developmental stage, solid food introduction, and suckling cessation.

## Introduction

The microbiota colonizes the mammalian intestine at birth, and its composition evolves with age towards an adult state, mainly under the influence of the diet. The gut microbiota is first shaped by components of maternal milk, such as fat, proteins, oligosaccharides, immunoglobulins, and antimicrobial peptides (1–3). Later in life, the introduction of solid food provides new, plant-derived substrates for the microbiota, which results in the growth of bacteria able to utilize these nutrients (4, 5).

The onset of solid food ingestion is also a critical step for the postnatal maturation of the intestinal epithelium. In the small intestine, an enzymatic switch occurs at the suckling-to-weaning transition with the decline of lactase involved in the digestion of milk lactose and the induction of disaccharidases involved in the terminal digestion of plant carbohydrates (6, 7). Key components of the epithelial defenses are also modulated around weaning, including tight-junctions, toll-like receptors (TLRs), antimicrobial peptides, and immunoglobulin transporters (4, 8–12). The maturation of the epithelium at the suckling-to-weaning transition is partly driven by hormonal and genetically wired factors (13–15). The timing of this developmental program governing epithelial maturation can be altered by modification of early life dietary intakes (6).

The gut microbiota also plays a pivotal role in the development of the intestinal epithelium at the onset of solid food ingestion (16–18). The microbial control of epithelial barrier maturation involves various mechanisms, including activation of TLR signaling and production of bacterial metabolites such as butyrate (4, 19). Moreover, bacterial signals can act on epithelial maturation at weaning, in combination with host-derived factors such as glucocorticoids (20). Conversely, the maturation of host immunity and metabolism during weaning also shapes the gut microbiota, notably through the modulation of epithelial innate immune responses and bile acid secretions (21, 22).

The comaturation of the gut microbiota and intestinal epithelium at the suckling-to-weaning transition is thus orchestrated by developmental signals and dietary intakes. Deciphering the relative contribution of these endogenous and nutritional factors has major implications for health, since the establishment of a symbiotic relationship between the microbiota and its host during a time window around weaning determines the long-term susceptibility to immune and metabolic diseases (23, 24). We hypothesized that the developmental stage, solid food ingestion, and suckling cessation contribute to drive the comaturation of the gut microbiota and intestinal epithelium at the suckling-to-weaning transition. Our objective was to decipher their respective contributions to this process.

Controlling early life dietary intakes is a technical challenge in rodent models, because maternal separation causes stress that disrupts intestinal homeostasis (25). Thus, the rabbit was selected as a model to study the comaturation of the gut microbiota and epithelium in mammals. Indeed, in this species, suckling occurs once a day for 5 minutes. After that, the mother leaves the nest and the pups are thus separated from the mother for the rest of the day (26). In experimental conditions, this characteristic allows for the precise control of milk and solid food ingestion by weighing the dam before and after suckling and weighing the pups’ feeders, which the dams cannot access, thus solely measuring the pups’ intake. Therefore, the rabbit model allowed us to accurately control milk and solid food availability at the suckling-to-weaning transition. We studied the development of the microbiota and epithelium in the cecum between postnatal day (PND) 18, which corresponds to the spontaneous onset of significant solid food ingestion, and PND25 in rabbits that were *1*) exclusively suckling; *2*) suckling and ingesting solid food; or *3*) exclusively ingesting solid food. The cecum was studied since this digestive segment is an important site of interaction between the microbiota and the digestive epithelium in rabbits (4). An analysis of the microbiota composition, metabolome, and epithelial gene expression highlighted the differential influences of the developmental stage, solid food introduction, and suckling cessation on the comaturation of the microbiota and epithelium at the suckling-to-weaning transition.

## Methods

### Animal experiments

The experiment was performed at the INRAE PECTOUL experimental facility (GenPhySE, INRAE). Animals were handled according to the European Union recommendations on the protection of animals used for scientific purpose (2010/63/EU) and in agreement with the French legislation (NOR: AGRG1238753A 2013). This protocol received the approval of the local ethics committee (Comité d’éthique en expérimentation animale SCIENCE ET SANTE ANIMALES N°115) (SSA_2019_004). The rabbits studied were crossbreeds from 2 commercial lines (maternal line: Hyplus PS19; paternal line: Hyplus PS59; Hypharm). Multiparous dams (*n* = 8) were housed individually in wire cages (61 × 69 × 49 cm) equipped with a closed nest for the pups (39 × 27 × 35 cm). The litter size was standardized to 10 pups per litter by cross-fostering at PND2.

**FIGURE 1:**
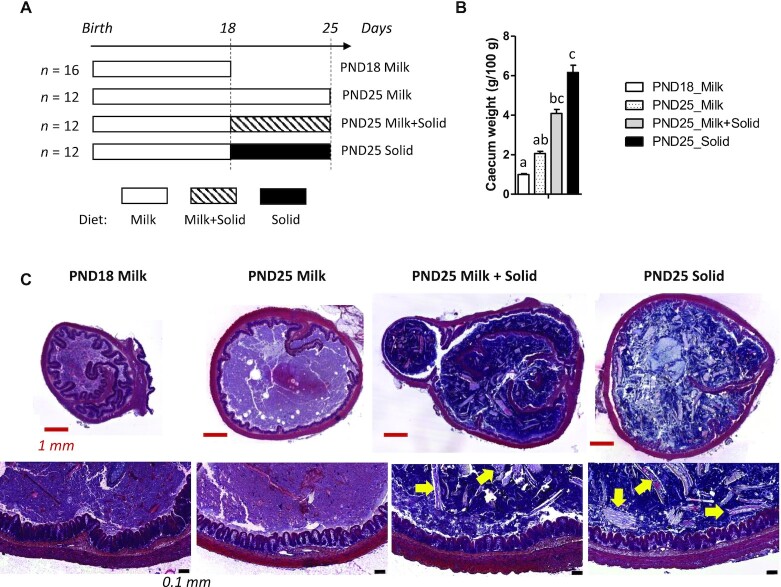
Influence of age and dietary intakes on rabbit cecum development. (A) Experimental design. (B) Cecum weight (tissue + content), expressed as a percentage of the live body weight. Bar plots show mean values and SEMs. The overall effects of experimental groups were tested with a Kruskal-Wallis test. Groups were compared pairwise with Wilcoxon tests. Labeled means without a common letter differ at a *P* value < 0.05. PND18 Milk: *n* = 16; PND25 Milk: *n* = 12; PND25 Milk + Solid: *n* = 12; and PND25 Solid: *n* = 12. (C) Hematoxylin and eosin–stained cecal sections were observed at low (upper row; red scale bars represent 1 mm) or high (lower row; black scale bars represent 0.1 mm) magnification. Yellow arrows indicate plant residues in the cecal content. Abbreviations: PND18 Milk, group of exclusively suckling rabbits at postnatal day 18; PND25 Milk, group of exclusively suckling rabbits at postnatal day 25; PND25 Milk + Solid, group of suckling rabbits ingesting solid food at postnatal day 25; PND25 Solid, group of rabbits ingesting exclusively solid food at postnatal day 25.

A schematic representation of the experimental design is presented in **Supplemental Figure 1**. All rabbit pups were exclusively suckling from birth to PND18. From this day onward, rabbits were divided into 3 groups (**Figure 1**A). In the first group (PND25 Milk; *n* = 4 litters with 1 litter/cage), as pups were exclusively suckling until PND25, the litter size was reduced to 5 pups/litter to ensure an adequate supply of milk. In the second group (PND25 Milk + Solid; *n* = 4 litters with 8 pups/litter and 1 litter/cage), pups were suckling and had ad libitum access to solid food pellets (StabiPro, Terrya). In the third group, rabbit pups (PND25 Solid; *n* = 12 pups from 4 litters in 2 cages) were weaned at PND18 and had ad libitum access to solid food pellets (StabiPro) but not to maternal milk. The chemical composition of the solid diet is shown in **Supplemental Table 1**. For suckling, the dam was placed once a day (for 5 minutes) in the nest of the pups. Milk ingestion by the litter was quantified at PND17 and PND24 by weighing the dam before and after suckling. Immediately after suckling, feces from the dam were removed from the nest to prevent coprophagia by the pups. The litter's solid food intake was quantified between PND21 and PND24 by weighing the feeder. The dam had no access to the feeder of the pups, and vice versa.

### Sample collection

Rabbit pups were euthanized by electronarcosis followed by exsanguination (Supplemental Figure 1). At PND18, 2 pups/litter (8 litters; *n* = 16; 8 females and 8 males) were euthanized after suckling (PND18 Milk). In the group of exclusively suckling rabbits (PND25 Milk), 3 pups/litter (4 litters; *n* = 12; 8 females and 4 males) were euthanized at PND25 after suckling. In the group of suckling rabbits with access to solid food pellets (PND25 Milk + Solid), 3 pups/litter (4 litters; *n* = 12; 6 females and 6 males) were euthanized at PND25 after suckling. In the group of early weaned rabbits (PND25 Solid; *n* = 12; 8 females and 4 males), all pups were euthanized at PND25. The cecum was isolated and weighted. The cecal content was collected and kept on ice until transfer to –80°C for long-term storage. The cecal content pH was measured with a glass electrode (VWR Collection SP225). One g of the cecal content was placed in 1 mL of 2% (w/v) H_2_SO_4_ for ammonia (NH_4_^+^) quantification with a colorimetric method using a continuous flow analyzer SAN++ (Skalar) as previously described (27). NH_4_^+^ and pH measurements could not be performed in some samples due to an insufficient amount of cecal content. For histological analyses, a section of cecal tissue with content was collected and placed in Carnoy's fixative solution (60% ethanol, 30% chloroform, 10% glacial acetic acid) for 3 hours before transfer in 70% ethanol and stored at 4°C. Cecal tissue fragments (4 × 2 cm) were washed in cold PBS and stored on ice until epithelial cell isolation.

### 16S ribosomal RNA gene amplicon sequencing and sequence analysis

Cecal content DNA was extracted using the Quick-DNA Fecal/Soil Microbe 96 Kit (ZymoResearch) and the 16S ribosomal RNA (rRNA) gene V3–V4 region was amplified by PCR and sequenced by MiSeq Illumina Sequencing as previously described (28). Sequencing reads were deposited in the National Center for Biotechnology Information Sequence Read Archive (SRA accession: PRJNA699723). The 16S rRNA gene amplicon sequences were analyzed using the Find, Rapidly, Operational taxonomic units with Galaxy Solution (FROGS) pipeline according to standard operating procedures (29). Amplicons were filtered according to their size (300–500 nucleotides) and clustered into operational taxonomic units (OTUs) using Swarm (aggregation distance: d = 1 + d = 3). After chimera removal, OTUs were kept when they were present in at least 3 samples and represented more than 0.005% of the total number of sequences (30). OTU affiliations were performed using the reference database silva138 16S with a minimum pintail quality of 100 (31). The mean number of reads per sample after filtering was 20,131 (minimum: 11,045; maximum: 37,579).

### Nuclear magnetic resonance metabolomics

The metabolome was analyzed in cecal content (50 mg) as previously described, using nuclear magnetic resonance at the MetaboHUB-MetaToul-AXIOM metabolomics platform, Toulouse, France (4). The results are presented as relative concentrations, with the PND18 Milk group used as a reference. Annotated representative spectra in each group are presented in **Supplemental Figure 2**. For each of the 29 identified metabolite, buckets not overlapping with other metabolites were selected for the quantification (**Supplemental Table 2**).

### Cecal epithelial cell isolation

Cecal tissue fragments stored in cold PBS were transferred in 5 mL of cold dissociation solution [9 mM EDTA (Thermo Fisher Scientific), 3 mM 1,4-Dithiothréitol (Roche) in PBS without Ca^2+^/Mg^2+^ (Thermo Fisher Scientific)]. After incubation (30 minutes at room temperature under agitation), cecal tissue was transferred in 5 mL cold PBS without Ca^2+^ and Mg^2+^. Epithelial crypts were detached by vigorous manual shaking (1 minute). After removal of the remaining cecal tissue, the presence of epithelial crypts in the solution was verified by brightfield microscopy (**Supplemental Figure 3**). After centrifugation (300 × *g* for 5 minutes at 4°C),  the supernatant was discarded and the crypt pellet was resuspended by vortexing in 300 μL cold TRI Reagent (ZymoResearch) before storage at –80°C until RNA extraction.

### Gene expression profiling in epithelial cells

After thawing, epithelial crypts lyzed by pipetting in TRI Reagent were vortexed before centrifugation (12,000 × *g* at 4°C for 10 minutes) to remove particles, and the supernatant was used for total RNA purification using the Direct-zol RNA MiniPrep Plus kit (ZymoResearch) and following the manufacturer's instructions, including a DNAse I treatment. The RNA concentration and purity were analyzed with a NanoDrop 8000 (Thermo Fisher Scientific). Complementary DNA were prepared from 500 ng RNA with the GoScript Reverse Transcription Mix, Random Primers kit (Promega) following the manufacturer's instructions. High-throughput real-time qPCR was performed using the Biomark microfluidic system using a 96.96 Dynamic Array IFC for gene expression (Fluidigm), according to the manufacturer's recommendations. The sequences of the primers used are presented in **Supplemental Table 3**. Data were analyzed with the 2^–ΔΔCt^ method with *GAPDH* gene expression used as a reference (32). The PND18 Milk group was used a reference for normalization.

### Histology

Transversal sections of cecal tissue with luminal content fixed in Carnoy's solution were embedded in paraffin and stained by hematoxylin and eosin in the histology platform Genotoul Anexplo. Slides were digitalized and the crypt depth (>8 well-oriented crypts/sample) was measured with the CaseViewer 2.3 software (3DHISTECH). The measurement was repeated twice by 2 independent investigators blinded to the groups.

### Immunoglobulin A quantification

Cecal content was diluted at 50 mg.mL^–1^in TBS buffer. After shaking thoroughly, the samples were centrifuged (3000 × *g* for 10 minutes at 4°C). The supernatants were collected and stored at –20°C until analysis. The total cecal IgA contents were determined in duplicate by sandwich ELISA using polyclonal goat anti-rabbit IgA antibodies (cat# A120-109P and A120-109A; Bethyl Laboratories). Sample dilution was adapted to experimental groups (suckling rabbits 1:1280 to 1:5120; weaned rabbits 1:320). Absorbance was measured at 450 nm with a GloMax Discover plate reader (Promega). Relative IgA concentrations were calculated using a standard curve obtained by serial dilution of a pool of all samples (rabbit IgA standard is not commercially available). IgA relative concentrations were normalized to the protein concentration in the cecal content measured with a colorimetric assay (Bio-Rad Protein Assay Dye Reagent Concentrate, Bio-Rad).

### Statistical analysis

All statistical analyses were performed using the R software (version 4.0.3; R Foundation for Statistical Computing, Vienna, Austria). The microbiota composition analysis was performed using the phyloseq package (version 1.26.1) (33). For α and β diversity analyses, the samples were rarefied to an even sequencing depth (11,045 reads per sample). The richness (observed OTUs) and Shannon α diversity were calculated. The β diversity was analyzed using the Bray-Curtis distance and plotted by nonmetric dimensional scaling. A permutational multivariate ANOVA was used to test the effects of groups on the Bray-Curtis distance between samples by using the vegan package (version 2.5–7). For the differential abundance analysis, OTUs representing less than 0.05% of the total number of sequences were filtered out. OTUs unrarefied counts were agglomerated at the phylum, family, or genus level, and relative abundances were calculated at each taxonomic level. Principal component analyses (PCAs) were performed with the mixOmics package on data obtained for metabolomics (relative abundance of 29 metabolites) and gene expression profiling (relative expression of 50 genes; version 6.14.0) (34). The heat map representation was created with the pheatmap package (version 1.0.12) using the Euclidean distance and Ward algorithm to cluster genes according to their relative expression.

Univariate statistical analyses to compare the 4 groups were performed with nonparametric Kruskal-Wallis tests, and the obtained *P* values were adjusted by the Benjamini-Hochberg method for bacterial groups (family and genus level) and metabolites. When the overall group effect was significant (*P* < 0.05), groups were compared pairwise using Wilcoxon tests with the Holm correction. No effect of sex was observed on any variate, and sex was thus not taken into account in statistical analyses. The effects of age were identified by comparison of exclusively suckling rabbits at PND18 and PND25 (PND18 Milk compared with PND25 Milk; Figure 1A). The effects of solid food introduction were identified by comparison at PND25 of exclusively suckling rabbits with rabbits that ingested milk and solid food (PND25 Milk compared with PND25 Milk + Solid). The effects of suckling cessation were identified by comparison at PND25 of rabbits that ingested milk and solid food with rabbits that ingested exclusively solid food (PND25 Milk + Solid compared with PND25 Solid).

## Results

Milk ingestion levels were similar at PND18 and PND25 in suckling rabbits consuming and not consuming solid food (mean: 35 g.pup^–1^.day^–1^; *n* = 8 litters at PND18 and *n* = 4 litters per group at PND25; **Supplemental Figure 4A**). At PND25, solid food ingestion was numerically 3-fold higher in rabbits consuming solid food only (PND25 Solid; *n* = 2 cages of 6 rabbits) when compared to rabbits consuming milk and solid food (PND25 Milk + Solid; *n* = 4 litters; 19.5 and 6.5 g.pup^–1^.day^–1^, respectively; Supplemental Figure 4B). When rabbits ingested both milk and solid food at PND25, solid food represented only 17% of the total fresh matter intake. The body weight of rabbits increased between PND18 and PND25 in all groups (Supplemental Figure 4C). The relative cecum weight increased between PND18 and PND25 only when rabbits ingested solid food (Figure 1B) and, as expected, plant-derived residues were observed in the cecal content only in these animals (Figure 1C).

### Influence of age and dietary intakes on the cecal microbiota diversity and structure

**FIGURE 2:**
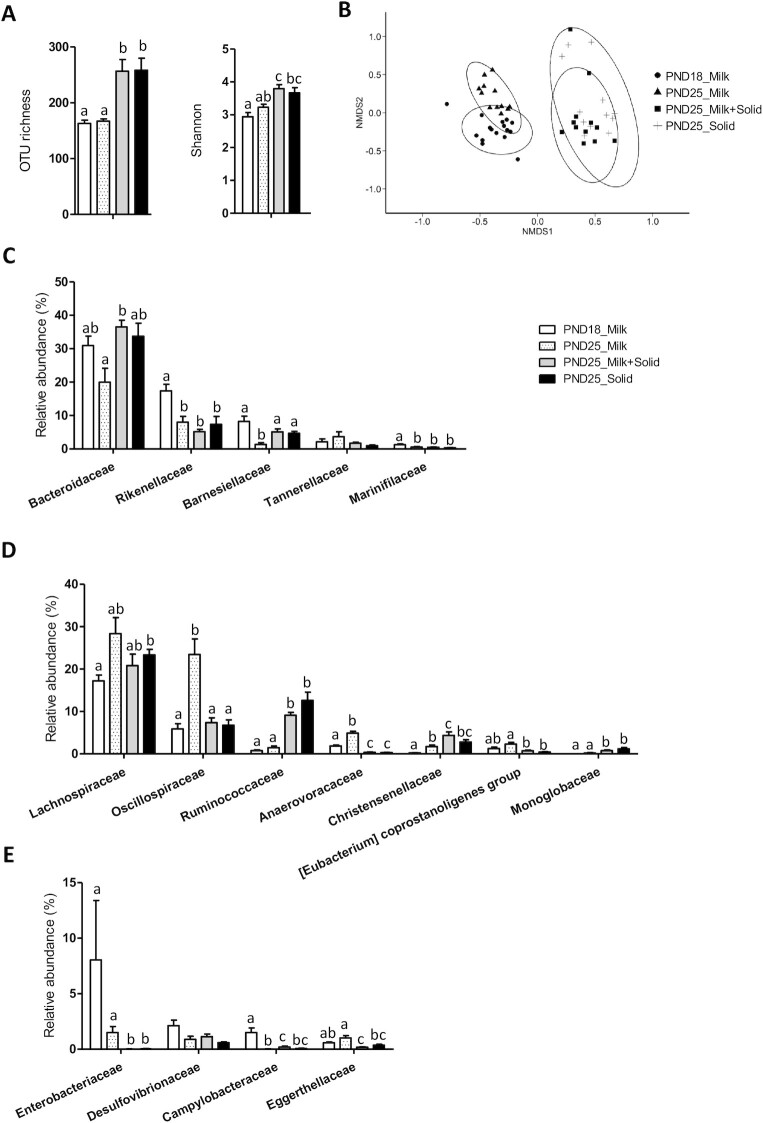
Influence of age and dietary intakes on rabbit cecum microbiota. (A) Microbiota richness and Shannon α diversity index. (B) Nonmetric dimensional scaling (nMDS) 2-dimensional representation of the microbiota β diversity using Bray-Curtis distance calculation (stress = 0.17). (C–E) Relative abundances of bacterial families are classified within the (C) Bacteroidota phylum, (D) Firmicutes phylum, and (E) other lower abundance phyla. (A, C–E) The bar plots show mean values and SEMs. The overall effects of experimental groups were tested with a Kruskal-Wallis test. Groups were compared pairwise with Wilcoxon tests. Labeled means without a common letter differ at a *P* value < 0.05. PND18 Milk: *n* = 16; PND25 Milk: *n* = 12; PND25 Milk + Solid: *n* = 12; and PND25 Solid: *n* = 12. Abbreviations: OTU, operational taxonomic unit; PND18 Milk, group of exclusively suckling rabbits at postnatal day 18; PND25 Milk, group of exclusively suckling rabbits at postnatal day 25; PND25 Milk + Solid, group of suckling rabbits ingesting solid food at postnatal day 25; PND25 Solid, group of rabbits ingesting exclusively solid food at postnatal day 25.

The relative abundance of each bacterial phylum, family, and genus are presented in **Supplemental Tables 4–6**. The β diversity analysis at the OTU level using the Bray-Curtis distance indicated that the microbiota structure was modified with age in exclusively suckling rabbits (PND18 Milk compared with PND25 Milk; explained variance *R*^2^ = 0.21; *P* < 0.001; **Figure 2**B). The relative abundances of Bacteroidota and families classified into this phylum (Rikenellaceae, Barnesiellaceae, Marinifilaceae) and Campylobacterota decreased with age, while the relative abundances of Firmicutes and families classified into this phylum (Oscillospiraceae, Anaerovoracaceae, Christensenellaceae) increased with age (Figure 2C–E; Supplemental Table 4). At PDN25, solid food ingestion by suckling rabbits increased OTU richness and Shannon α diversity and strongly modified the microbiota structure (PND25 Milk compared with PND25 Milk + Solid; explained variance *R*^2^ = 0.26; *P* < 0.001; Figure 2A and B). The introduction of solid food prevented the age-related increases of Oscillospiraceae and Anaerovoracaceae and the age-related decrease of Barnesiellaceae (Figure 2C and D). In contrast, solid food introduction amplified the increased relative abundance of Christensenellaceae observed with age (Figure 2D). The introduction of solid food also increased the relative abundances of Ruminococcaceae and Monoglobaceae, while it reduced the relative abundances of Enterobacteriaceae and Eggerthellaceae (Figure 2D and E). Strikingly, suckling cessation in rabbits ingesting solid food had no effect on the microbiota diversity and structure (PND25 Milk + Solid compared with PND25 Solid). Altogether, our results indicate that the microbiota was mainly influenced by solid food introduction and, to a lesser extent, by age, while suckling cessation had no effect.

### Influence of age and dietary intakes on the cecal metabolome

**FIGURE 3:**
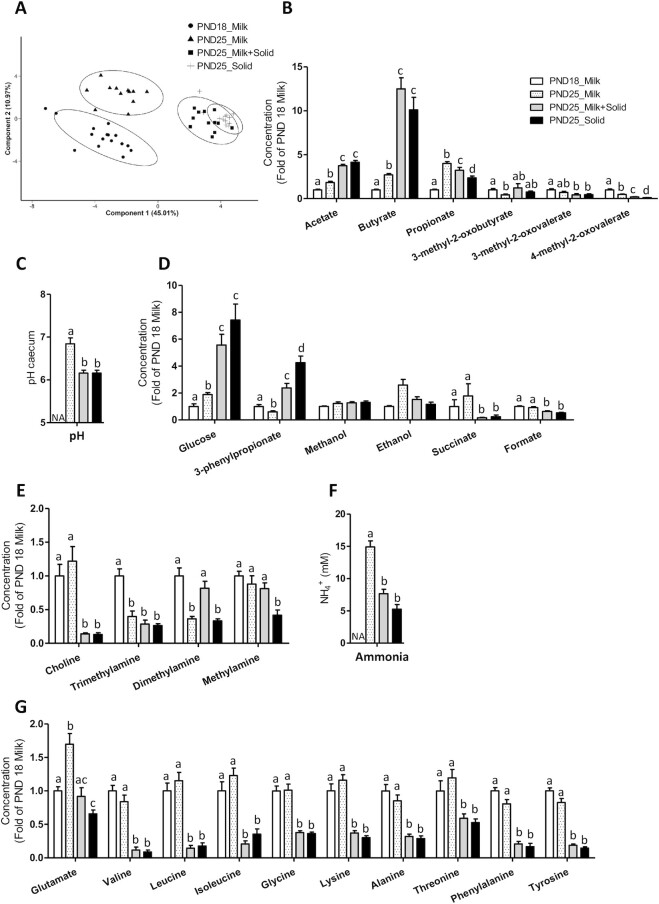
Influence of age and dietary intakes on rabbit cecum metabolome. (A) Individual plot of PCA of metabolites. (B) Relative concentration of SCFAs. (C) Cecal content pH. The measure was not performed at PND18 due to an insufficient amount of cecal content. (D) Relative concentrations of miscellaneous metabolites. (E) Relative concentrations of choline and of its microbial catabolites. (F) Ammonia (NH_4_^+^) concentrations in cecal content. The measure was not performed at PND18 due to an insufficient amount of cecal content. (G) Relative concentrations of amino acids. (B–G) The bar plots show mean values and SEMs. The overall effects of experimental groups were tested with a Kruskal-Wallis test. Groups were compared pairwise with Wilcoxon tests. Labeled means without a common letter differ at a *P* value < 0.05. PND18 Milk: *n* = 16; PND25 Milk: *n* = 12; PND25 Milk + Solid: *n* = 12; and PND25 Solid: *n* = 12. Abbreviations: PCA, principal component analysis; PND18 Milk, group of exclusively suckling rabbits at postnatal day 18; PND25 Milk, group of exclusively suckling rabbits at postnatal day 25; PND25 Milk + Solid, group of suckling rabbits ingesting solid food at postnatal day 25; PND25 Solid, group of rabbits ingesting exclusively solid food at postnatal day 25.

PCA indicated that the cecal metabolome was mainly influenced by the introduction of solid food (PCA axis 1; 45% of variance) and by age (PCA axis 2; 11% of variance; **Figure 3**A; **Supplemental Table 7**). The relative concentrations of acetate, butyrate, propionate, glucose, and glutamate increased with age in exclusively suckling rabbits, while the relative concentrations of 3-methyl-2-oxobutyrate, 4-methyl-2-oxovalerate, 3-phenylpropionate, trimethylamine, and dimethylamine decreased with age (PND18 Milk compared with PND25 Milk; Figure 3B, D, E, and G). Solid food ingestion by suckling rabbits amplified the age-related increases of acetate, butyrate, and glucose and the age-related decrease of 4-methyl-2-oxovalerate (PND25 Milk compared with PND25 Milk + Solid; Figure 3B and D). In contrast, solid food introduction reduced the age-related increase of propionate (Figure 3B). Solid food introduction had the opposite effect of age on 3-phenylpropionate, since it strongly increased its relative concentration (Figure 3D). The introduction of solid food also decreased the cecal pH and the relative concentrations of succinate, formate, choline, ammonia, and amino acids (Figure 3C–G). Suckling cessation in rabbits ingesting solid food reduced the relative concentrations of propionate, 4-methyl-2-oxovalerate, and methylamine, while it increased the relative concentration of 3-phenylpropionate (PND25 Milk + Solid compared with PND25 Solid; Figure 3A, D and E). Taken together, our metabolomics data indicated that the changes of the gut microbiota metabolic activity at the suckling-to-weaning transition were mainly driven by solid food introduction and, to a lesser extent, by age and by suckling cessation.

### Influence of age and dietary intakes on gene expression in the cecal epithelium

**FIGURE 4:**
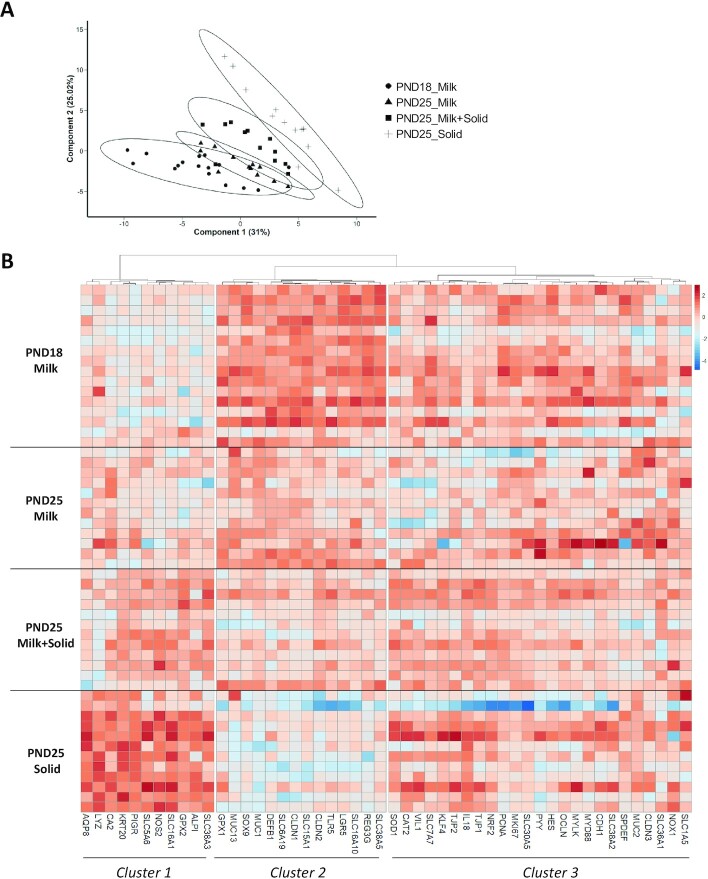
Influence of age and dietary intakes on gene expression in rabbit cecum. (A) Individual plot of PCA of gene expression. (B) Heat map representing the relative expression of genes (columns) in individual samples (rows). The colors represent the Z-scores (row-scaled relative concentration) from low (blue) to high values (red). Genes (columns) were clustered by the Ward method. PND18 Milk: *n* = 16; PND25 Milk: *n* = 12; PND25 Milk + Solid: *n* = 12; and PND25 Solid: *n* = 12. Abbreviations: PCA, principal component analysis; PND18 Milk, group of exclusively suckling rabbits at postnatal day 18; PND25 Milk, group of exclusively suckling rabbits at postnatal day 25; PND25 Milk + Solid, group of suckling rabbits ingesting solid food at postnatal day 25; PND25 Solid, group of rabbits ingesting exclusively solid food at postnatal day 25.

PCA revealed that gene expression in the cecal epithelium was influenced by age, solid food introduction, and suckling cessation (**Figure 4**A). We identified a first cluster of genes (Cluster 1) whose expression increased with a cumulative effect of age, solid food introduction, and suckling cessation (Figure 4B). This cluster included genes coding for epithelial differentiation markers (*KRT20,CA2,AQP8,ALPI*), mediators of epithelial defenses [lysozyme (*LYZ*), the polymeric immunoglobulin receptor (*PIGR*), nitric oxide synthase (*NOS2*), glutathione-peroxidase 2 (*GPX2*)], and nutrient transporters (*SLC16A1,SLC38A3*). In contrast, we identified a second cluster of genes (Cluster 2) whose expression decreased with a cumulative effect of age, solid food introduction, and suckling cessation (Figure 4B). This cluster included genes coding for epithelial stem cell markers (*LGR5,SOX9*), mediators of epithelial innate immunity [*REG3G*, defensin beta 1 (*DEFB1*), *TLR5,GPX1*], epithelial barrier components [*MUC1,MUC13*, and tight junction proteins claudin 1 and 2 (*CLDN1* and *CLDN2*, respectively)], and amino acid transporters (*SLC6A19,SLC15A1,SLC16A10,SLC38A5*).

### Epithelial transport of nutrients

**FIGURE 5:**
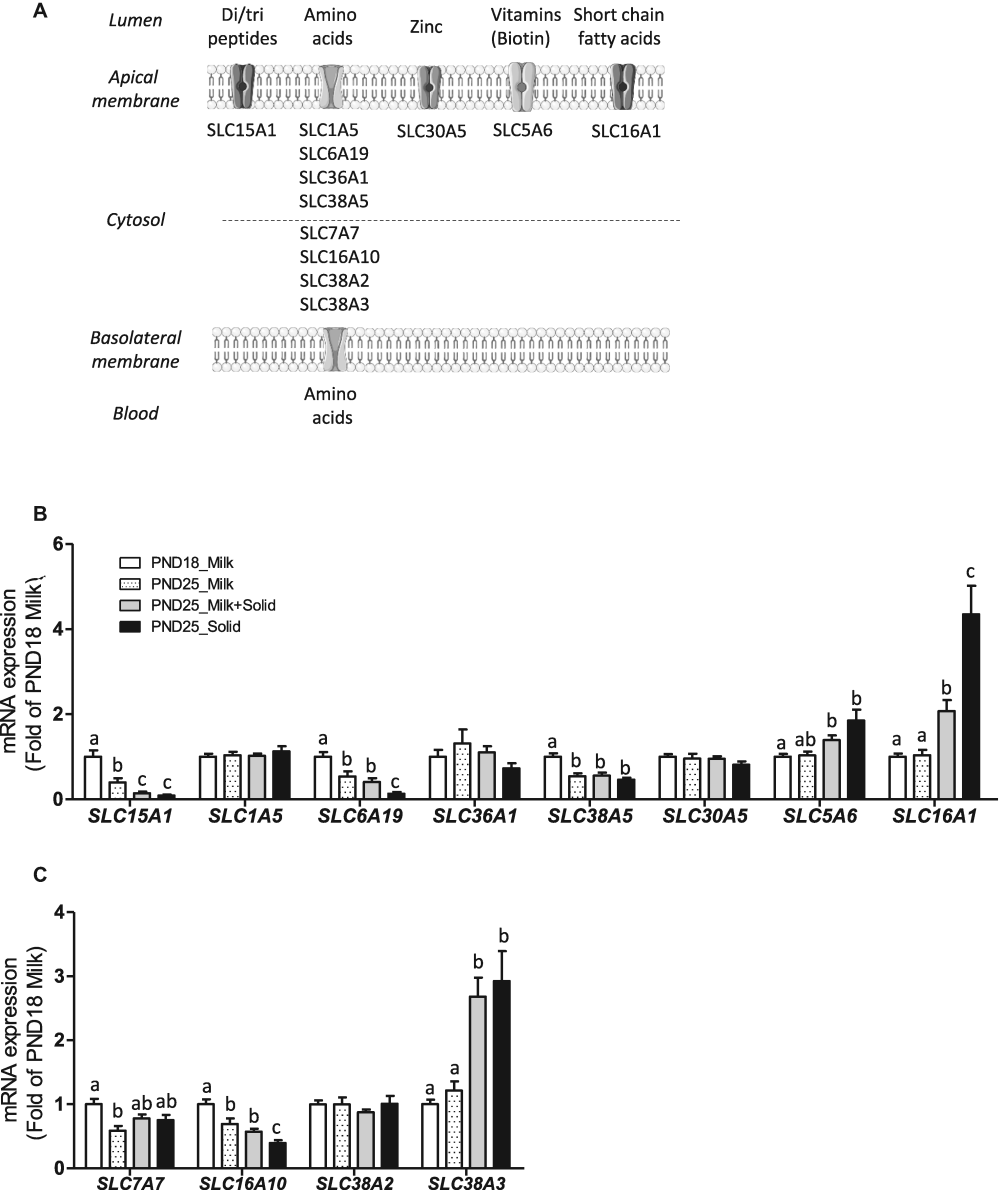
Influence of age and dietary intakes on epithelial nutrient transport in rabbit cecum. (A) Schematic representation of the cellular localization of the nutrient transporter in intestinal epithelial cells (created with BioRender.com). Relative mRNA levels of (B) apical and (C) basolateral nutrient transporters. The bar plots show mean values and SEMs. The overall effects of experimental groups were tested with a Kruskal-Wallis test. Groups were compared pairwise with Wilcoxon tests. Labeled means without a common letter differ at a *P* value < 0.05. PND18 Milk: *n* = 16; PND25 Milk: *n* = 12; PND25 Milk + Solid: *n* = 12; and PND25 Solid: *n* = 12. Abbreviations: PND18 Milk, group of exclusively suckling rabbits at postnatal day 18; PND25 Milk, group of exclusively suckling rabbits at postnatal day 25; PND25 Milk + Solid, group of suckling rabbits ingesting solid food at postnatal day 25; PND25 Solid, group of rabbits ingesting exclusively solid food at postnatal day 25.

The gene expression of peptide and amino acid transporters (*SLC15A1,SLC6A19,SLC38A5,SLC7A7,SLC16A10*) was downregulated with age in exclusively suckling rabbits (PND18 Milk compared with PND25 Milk; **Figure 5**A–C). Ingestion of solid food by suckling rabbits amplified the downregulation of the peptide transporter *SLC15A1*, while it upregulated the expression of transporters for amino acids *SLC38A3* and SCFAs (*SLC16A1*; PND25 Milk compared with PND25 Milk + Solid; Figure 5A–C). Suckling cessation in rabbits ingesting solid food amplified the downregulation of amino acid transporters (*SLC6A19* and *SLC16A10*) and the upregulation of the SCFA transporter *SLC16A1* (PND25 Milk + Solid compared with PND25 Solid; Figure 5B and C).

### Epithelial renewal and differentiation

**FIGURE 6:**
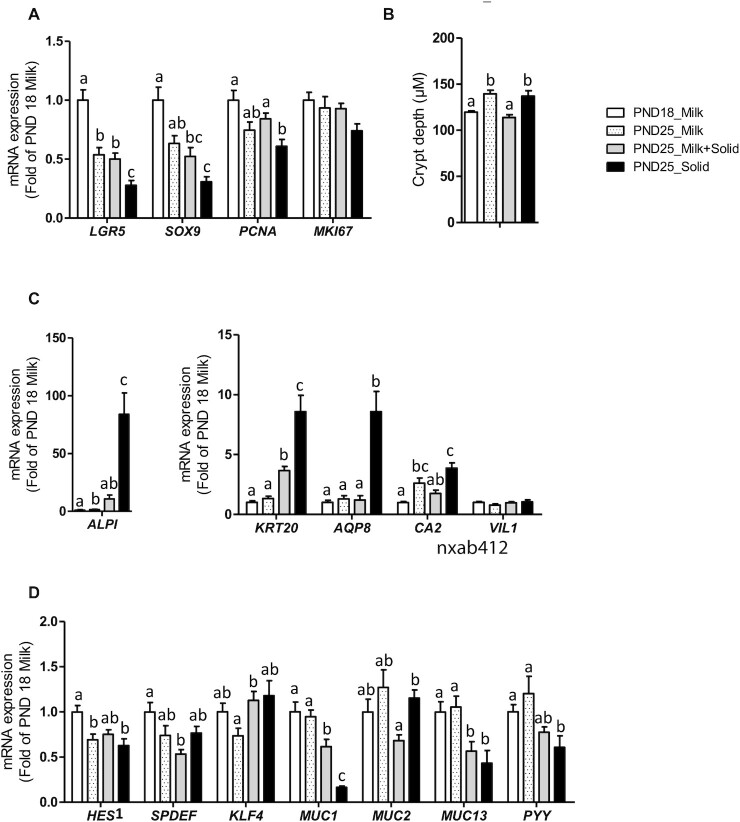
Influence of age and dietary intakes on epithelium renewal and differentiation in rabbit cecum. (A) Relative mRNA levels of stem cell and proliferation markers. (B) Cecal epithelial crypt depth. (C) Relative mRNA levels of epithelial differentiation and absorptive cell markers. (D) Relative mRNA levels of secretory cell (goblet and enteroendocrine) markers. (A–D) The bar plots show mean values and SEMs. The overall effects of experimental groups were tested with a Kruskal-Wallis test. Groups were compared pairwise with Wilcoxon tests. Labeled means without a common letter differ at a *P* value < 0.05. PND18 Milk: *n* = 16; PND25 Milk: *n* = 12; PND25 Milk + Solid: *n* = 12; and PND25 Solid: *n* = 12. Abbreviations: PND18 Milk, group of exclusively suckling rabbits at postnatal day 18; PND25 Milk, group of exclusively suckling rabbits at postnatal day 25; PND25 Milk + Solid, group of suckling rabbits ingesting solid food at postnatal day 25; PND25 Solid, group of rabbits ingesting exclusively solid food at postnatal day 25.

The gene expression of the stem cell marker *LGR5* and of *HES1* (a transcription factor repressing secretory lineage commitment) were reduced with age in exclusively suckling rabbits, while the gene expression of absorptive cell markers (*ALPI* and *CA2*) increased with age (PND18 Milk compared with PND25 Milk; **Figure 6**A–D). Ingestion of solid food by suckling rabbits reduced the expression of transmembrane mucins (*MUC1* and *MUC13*), while it upregulated the gene expression of the differentiation maker *KRT20* and of *KLF4*, a transcription factor involved in goblet cell differentiation (PND25 Milk compared with PND25 Milk + Solid; Figure 6C and D). Suckling cessation in rabbits ingesting solid food amplified the downregulation of *LGR5* and *MUC1* and the upregulation of *ALPI* and *KRT20* (PND25 Milk + Solid compared with PND25 Solid; Figure 6A, C, and D). Suckling cessation also downregulated the expression of the proliferation marker *PCNA* and upregulated the expression of the absorptive cell marker *AQP8* (Figure 6A and C). Epithelial crypt depth increased between PND18 and PND25 when rabbits ingested either only solid food or only milk but, surprisingly, not when they ingested both solid food and milk (Figure 6B).

### Epithelial barrier

**FIGURE 7:**
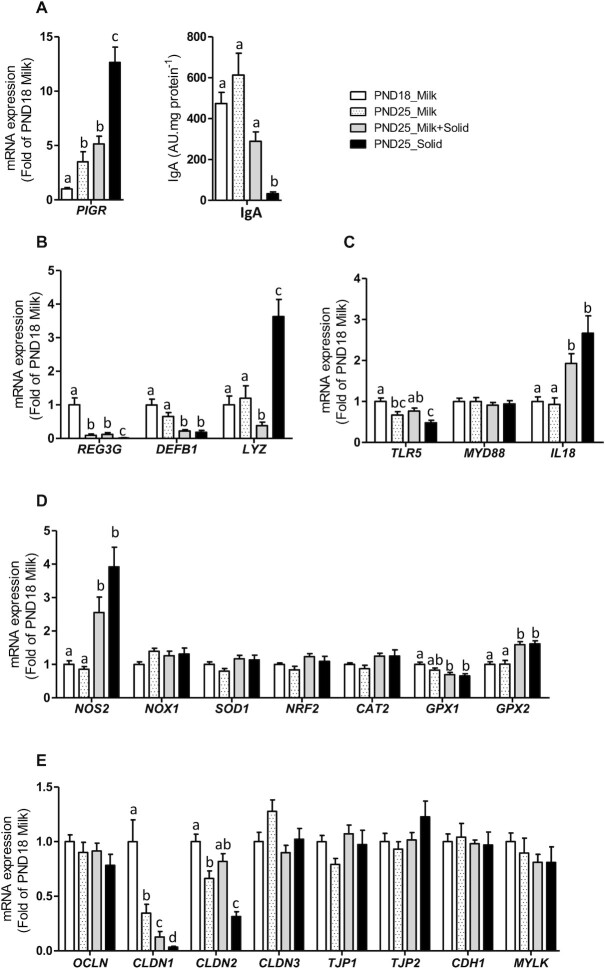
Influence of age and dietary intakes on epithelial barrier functions in rabbit cecum. (A) Relative mRNA level of the polymeric immunoglobulin transporter. Immunoglobulin A was quantified in cecal content by ELISA. The results were normalized by the protein concentration in the cecal content. (B–E) Relative mRNA levels of (B) antimicrobial peptides, (C) proteins involved in microbial sensing, (D) redox homeostasis, and (E) epithelial junctions. (A–E) The bar plots show mean values and SEMs. The overall effects of experimental groups were tested with a Kruskal-Wallis test. Groups were compared pairwise with Wilcoxon tests. Labeled means without a common letter differ at a *P* value < 0.05. PND18 Milk: *n* = 16; PND25 Milk: *n* = 12; PND25 Milk + Solid: *n* = 12; and PND25 Solid: *n* = 12. Abbreviations: AU, arbitrary units; PND18 Milk, group of exclusively suckling rabbits at postnatal day 18; PND25 Milk, group of exclusively suckling rabbits at postnatal day 25; PND25 Milk + Solid, group of suckling rabbits ingesting solid food at postnatal day 25; PND25 Solid, group of rabbits ingesting exclusively solid food at postnatal day 25.

The expression of the *PIGR* increased with age in exclusively suckling rabbits, while the expression of the antimicrobial peptide *REG3G, TLR5*, and *CLDN1* and *CLDN2* decreased with age (PND18 Milk compared with PND25 Milk; **Figure 7**A–C and E). Ingestion of solid food by suckling rabbits amplified the downregulation of *CLDN1* (PND25 Milk compared with PND25 Milk + Solid; Figure 7E). The introduction of solid food also upregulated the gene expression of the cytokine *IL18*, the prooxidant enzyme inducible *NOS2*, and the anti-oxidant enzyme *GPX2* while it downregulated the expression of the antimicrobial peptides *LYZ* and *DEFB1* (Figure 7B–D). Suckling cessation in rabbits ingesting solid food amplified the upregulation of *PIGR*, and this effect was associated with a strong reduction of the IgA concentration in the cecum (PND25 Milk + Solid compared with PND25 Solid; Figure 7A). Suckling cessation also amplified the downregulation of *REG3G* and *TLR5*, while it strongly upregulated the gene expression of *LYZ*(Figure 7B and C). Overall, gene expression profiling indicates that the maturation of the intestinal epithelium at the suckling-to-weaning transition was influenced by the combination of age, solid food introduction, and suckling cessation.

## Discussion

The suckling-to-weaning transition is considered to be a window of opportunity for the development of a mutualistic relationship between the gut microbiota and its host, with long-term consequences for health (23, 24). Indeed, the gut microbiota composition and metabolic activity are highly remodeled at the onset of solid food ingestion, which coincides with the maturation of the intestinal epithelium (4, 9, 35). However, deciphering the relative contributions of endogenous and dietary factors driving this developmental process is an experimental challenge. Here, we took advantage of the unique suckling behavior of rabbits (mother-pup separation except for 5 minutes once a day) to delineate the influence of the developmental stage (age), solid food introduction, and suckling cessation on the comaturation of the gut microbiota and epithelium at the suckling-to-weaning transition.

### Influence of age in exclusively suckling rabbits

The slight modification of the microbiota structure with age in exclusively suckling rabbits is in agreement with a previous study using the same model (36). By using culture-based methods, the authors observed a reduction of the Colibacilli flora with age in the cecum, although only maternal milk was ingested (36). The age-related regulation of epithelial innate immunity observed in our study (e.g., downregulation of the gene expression of REG3G and TLR5) could contribute to the modification of the microbiota composition with age in exclusively suckling rabbits. Indeed, the age-dependent modification of TLR5 expression was shown in mice to influence the microbiota composition through the antimicrobial peptide REG3G (22). Changes in milk composition according to lactation stage (PND18 compared with PND25) could also influence the gut microbiota. For instance, the milk oligosaccharide composition changes during lactation in humans (37). However, the variation in the milk oligosaccharide composition according to lactation stage has not been described in rabbits. Importantly, the developmental stage did not modify the microbiota towards a solid food–oriented microbiota that can be considered as more mature. The age-related increase in concentrations of the bacterial metabolites acetate, propionate, and butyrate could be related to an increased availability for the microbiota of substrates that can be metabolized into SCFA, either derived from milk (e.g., oligosaccharides) or from the host (38). The important increase with age of propionate (4-fold) is in agreement with previous results obtained in exclusively suckling rabbits (36).

The age-related decline in expression of amino acid transporters in the epithelium is consistent with previous studies showing a gradual reduction of amino acid transport from birth to weaning in mammals, including rabbits (39). This effect was observed despite the absence of change in the relative concentration of amino acids in the lumen, suggesting that the regulation of these transporters is driven by an intrinsic developmental program corresponding to the capacity of the large intestine to absorb peptides and amino acids, mostly in early life (40). Indeed, ontogenic factors (e.g., hormonal status) are thought to play an important role in the maturation of the intestinal epithelium (6, 7). Endogenous factors could also be involved in the upregulation of *PIGR* expression that we observed with age in exclusively suckling rabbits, independently of modifications of cecal IgA concentrations. Previous studies in mice demonstrated that the upregulation of PIGR at weaning is driven by hormonal modifications during this developmental transition (41). The downregulation of the gene expression of the epithelial stem cell marker LGR5 (leucine-rich repeat-containing G-protein coupled receptor 5) with age in exclusively suckling rabbits could be linked to a decreased concentration of milk growth factors, as observed before weaning in mice (42). Our results also showed that developmental factors play a major role in the decline of the gene expression of epithelial junction proteins (CLDN1 and CLDN2) observed at the suckling-to-weaning transition in rabbits (4). These results probably reflect the important synthesis of tight junction proteins in the developing cecum that gradually decreases as maturation progresses.

Altogether, our results show that age influences the gut microbiota and gene expression in the epithelium. Ontogenic factors (e.g., hormonal status) and modification of the milk composition according to lactation stage (e.g., oligosaccharides, growth hormones) probably play an important role in these effects.

### Influence of solid food ingestion in suckling rabbits

The major effect of solid food introduction on the gut microbiota richness (∼1.5-fold increase) and structure can be linked to the presence in the cecum content of new substrates derived from plants. In agreement with this hypothesis, solid food introduction induced a bloom in Ruminococcaceae (10-fold increase), which is a bacterial family specializing in complex plant polysaccharides degradation that typically blooms after weaning in humans, pigs, and rabbits (4, 43–45). Primary degradation of plant carbohydrates by the microbiota probably explains the increased concentration of glucose observed in the cecum after the introduction of solid food (46). Subsequent glucose fermentation by the microbiota might explain the increased concentration in SCFA induced by introducing solid food, as observed in previous studies (4, 46–48). This increased production of SCFA by the microbiota could be responsible for the increased gene expression of the epithelial transporter of SCFA (*SLC16A1*/MCT1), since its expression is upregulated by butyrate (49). The increased concentration of butyrate could also contribute to the prodifferentiation effect of the introduction of solid food (e.g., upregulation of *KRT20* expression), since this bacterial metabolite is known to promote epithelial differentiation (50).

The increased concentration of the polyphenol-derived bacterial metabolite 3-phenylpropionate (hydrocinnamic acid) upon introduction of solid food again suggested regulation of the metabolic activity of the microbiota by plant-derived substrates (51). In contrast, the reduction in the microbial amino acid catabolic product ammonia after the introduction of solid food can be linked to the low concentration of amino acids in the cecal lumen (52).

The remodeling of the microbiota composition induced by the introduction of solid food probably triggers the upregulation of the gene expression of the cytokine IL-18 and of the enzymes involved in epithelial redox homeostasis, NOS2 and GPX2. Indeed, the colonization of the gut by the microbiota is required for the upregulation of NOS2 at weaning in mice (17). In turn, this upregulation of epithelial innate immune responses could also shape the microbiota composition (53). Importantly, the major influence of introducing solid food on the microbiota and epithelium was observed despite the quantity of solid food ingested being much lower that the quantity of milk ingested. This observation suggests that the introduction of a small amount of solid food is sufficient to trigger comaturation of the microbiota and epithelium in suckling mammals.

### Influence of suckling cessation in rabbits ingesting solid food

The absence of effects of suckling cessation on the microbiota diversity and structure suggests that once solid food has been ingested, it overwhelms the influence of milk components that are known to shape the microbiota (e.g., oligosaccharides, IgA, innate immune factors) (1, 3). In contrast with our results, longitudinal studies in humans suggested that the maturation of the gut microbiota was driven by cessation of breastfeeding rather than by the introduction of solid foods (54, 55). However, in these human studies, the effects of the cessation of breastfeeding and solid food introduction cannot be completely disentangled due to gradual dietary transitions (5). In comparison to the microbiota, the effects of suckling cessation were more pronounced at the epithelial level. The downregulation of stem cell and proliferation markers after suckling cessation is consistent with a role for milk growth hormones in epithelial proliferation (6). The loss of milk hormones could also contribute to the strong prodifferentiation effect of suckling cessation. Alternatively, the stress potentially caused by the early weaning procedure could result in an increased glucocorticoid level, which is known to enhance epithelial differentiation (7). The strong upregulation of *PIGR* expression induced by suckling cessation could be due to the loss of maternal transfer of immunoglobulins (i.e., passive immunity), as demonstrated in mouse studies (10). Indeed, very low IgA levels were measured in rabbit cecum after suckling cessation, which is consistent with the maternal origin of IgA before weaning (i.e., passive immunity) (56). Interestingly, most of the effects of suckling cessation at the epithelial level corresponded to an amplification of the effect of age. This observation is consistent with the idea that dietary modification can accelerate but not override the genetically wired development of the epithelium (6, 7). Altogether, our results suggest that the loss of milk-derived molecules (e.g., growth hormones, IgA) after suckling cessation has no effect on the microbiota, while it probably accounts for a major part of the effect on the epithelium development.

## Conclusion

Our results show that the gut microbiota maturation at the suckling-to-weaning transition is mainly driven by the introduction of solid food, and to a lesser extent by age, while suckling cessation had no effect. Solid food introduction was also the main factor influencing the production of metabolites by the microbiota, while the influences of age and suckling cessation were lower. Complex interactions between the developmental stage, solid food introduction, and suckling cessation were involved in the maturation of the intestinal epithelium. Additional studies are required to determine the optimal timing of solid food introduction and its nutritional composition in order to promote the comaturation of the gut microbiota and epithelium with the aim of promoting long-term health (57).

## Supplementary Material

nxab411_Supplemental_File

## Data Availability

Data described in the manuscript will be made available upon request. Sequencing reads were deposited in the National Center for Biotechnology Information Sequence Read Archive (SRA accession: PRJNA699723).

## Notes

This work was supported by the ICSA (“eFeedIt” project).

Author disclosures: The authors report no conflicts of interest.

The supporting source had no involvement or restrictions regarding publication.

Supplemental Figures 1–4 and Supplemental Tables 1–7 are available from the “Supplementary data” link in the online posting of the article and from the same link in the online table of contents at https://academic.oup.com/jn/.

Abbreviations used: *CLDN1*, claudin 1; *CLDN2*, claudin 2; GPX2, glutathione-peroxidase 2; *LYZ*, lysozyme; *NOS2*, nitric oxide synthase; PCA, principal component analysis; *PIGR*, polymeric immunoglobulin receptor; PND, postnatal day; PND18 Milk, group of exclusively suckling rabbits at postnatal day 18; PND25 Milk, group of exclusively suckling rabbits at postnatal day 25; PND25 Milk + Solid, group of suckling rabbits ingesting solid food at postnatal day 25; PND25 Solid, group of rabbits ingesting exclusively solid food at postnatal day 25; rRNA, ribosomal RNA; TLR, toll-like receptor.
