# Supplementary material for: Developmental Stage, Solid Food Introduction, and Suckling Cessation Differentially Influence the Comaturation of the Gut Microbiota and Intestinal Epithelium in Rabbits
Source: J Nutr. 2021 Dec 7;152(3):723–36. doi: 10.1093/jn/nxab411 (PMC8891179; doi:10.1093/jn/nxab411)
Supplement: nxab411_Supplemental_File [file nxab411_supplemental_file.docx]

**
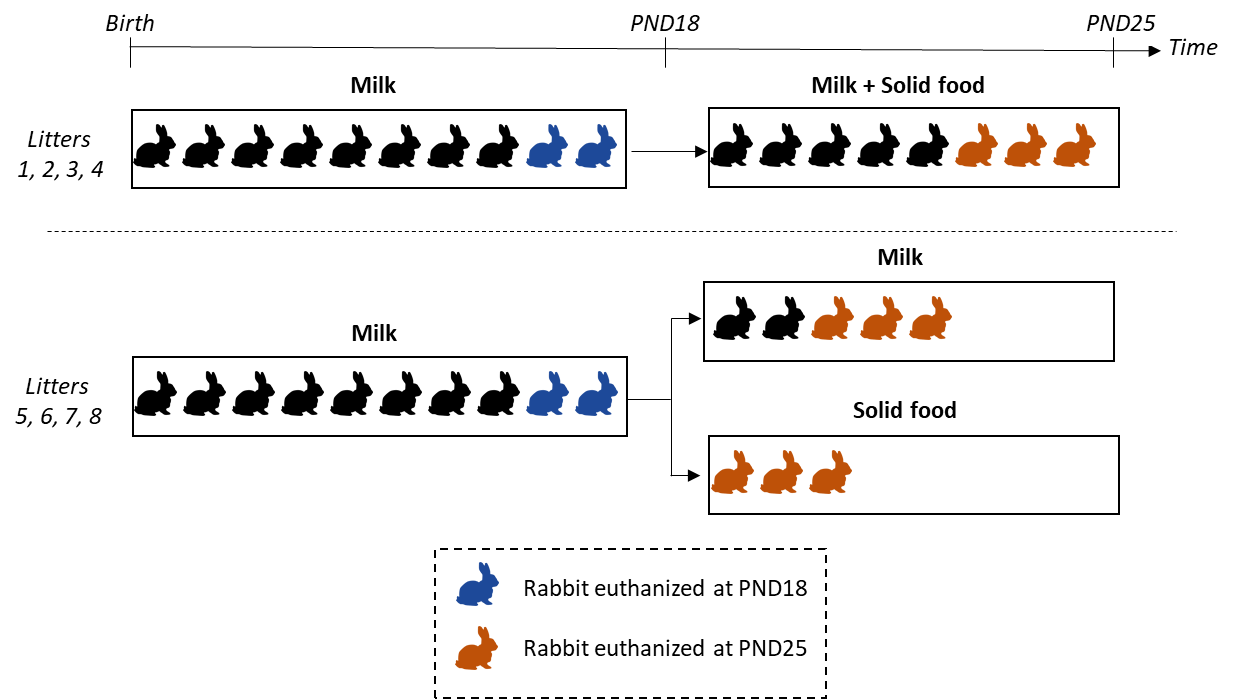
**

**Supplementary figure 1: Schematic representation of the experimental design**. From birth to postnatal day 18 (PND18), 10 rabbit pups per litter (8 litters in total) were exclusively suckling. Two rabbit pups in each litter were euthanized at PND18 (depicted in blue, PND18 Milk group, total n=16 pups). At PND18, the litters were separated in two groups. In four litters (#1, 2, 3, 4), the eight remaining rabbit pups were suckling and had access to solid food until PND25. Three rabbit pups from these litters were euthanized at PND25 (depicted in orange, PND25 Milk+Solid group, total n=12 pups). In the four other litters (#5, 6, 7, 8), three rabbits were housed in a separate cage and had access to solid food only at PDN25. These three rabbit pups were euthanized at PND25 (depicted in orange, PND25 Solid group, total n=12). The remaining five rabbits were exclusively suckling until PND25. Three of these pups were euthanized at PND25 (depicted in orange, PND25 Milk group, total n=12).

**
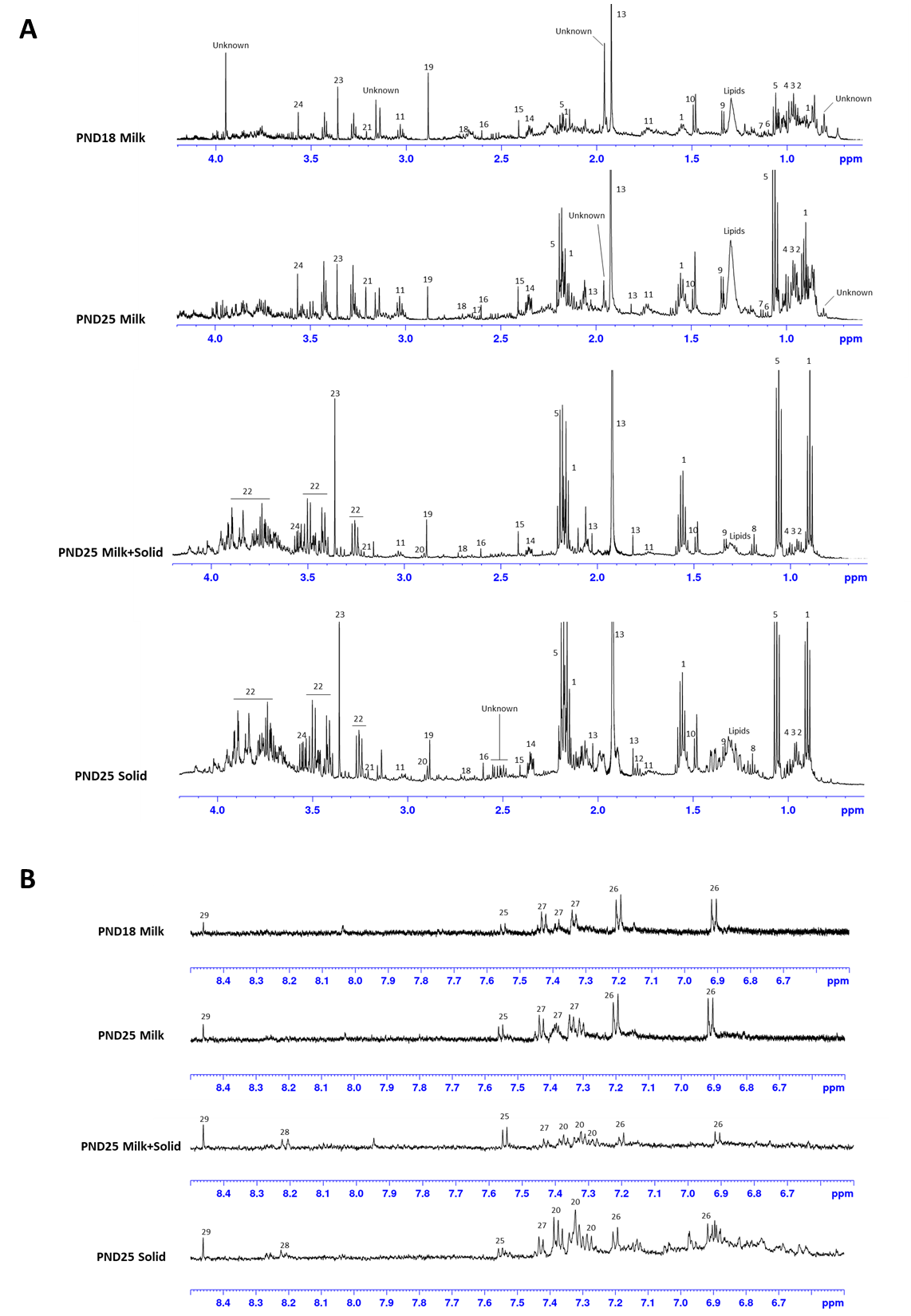
**

**Supplementary figure 2:** Aliphatic region (δ^1^H 4.20 – 0.6 ppm) (A) and aromatic region (δ^1^H 8.5 – 6.5 ppm) (B) of the NMR spectra of the caecal contents from one rabbit of each group (PND18 Milk, PND25 Milk, PND25 Milk+Solid, PND25 Solid). PND: Postnatal day. Numbers indicate identified metabolites described in supplementary table 2.

**
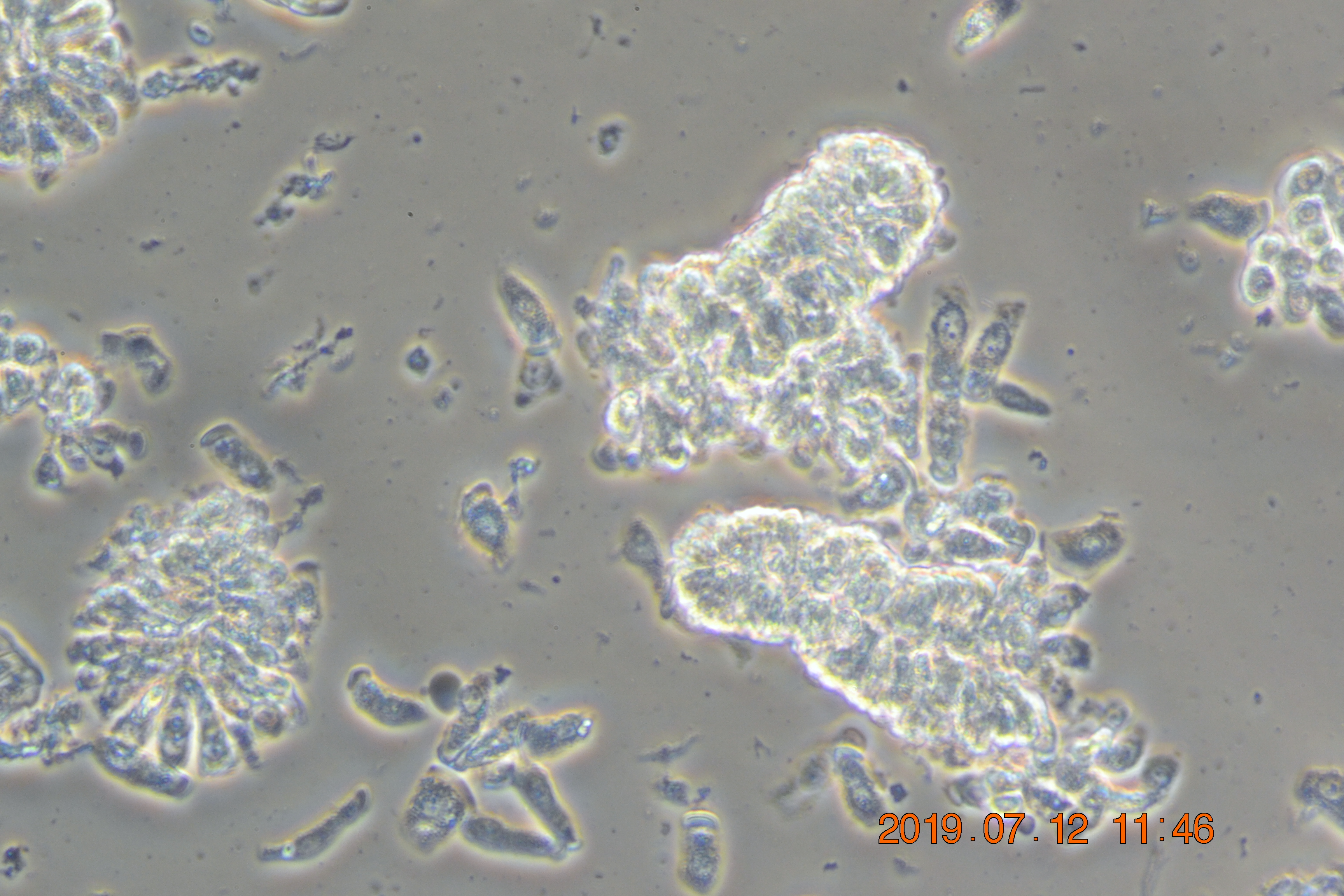
**

**Supplementary figure 3:** Brightfield microscopy observation (100x) of rabbit caecal epithelial crypts used for RNA extraction.

**
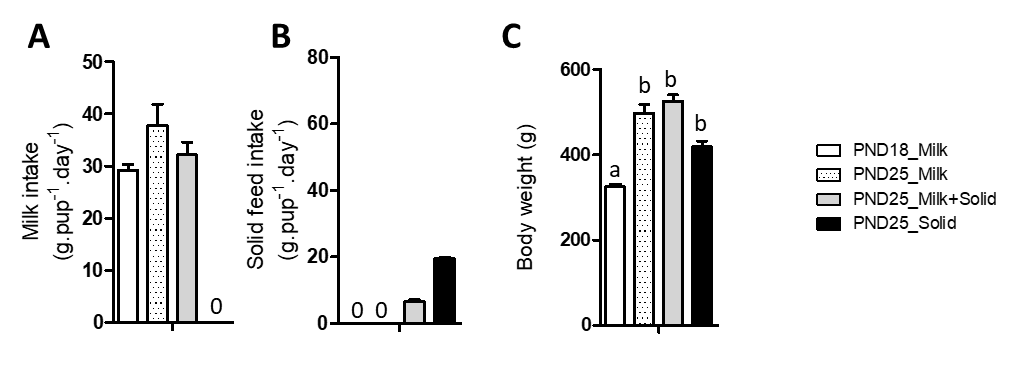
**

**Supplementary figure 4: Dietary intakes and growth of rabbits**. A - Milk intake (fresh matter) per rabbit pup was measured in each litter at PND17 (group PND18 Milk, n=8 litters) or PND24 (groups PND25 Milk and PND25 Milk+Solid, n=4 litters). B - Solid food intake (fresh matter) per rabbit pup per day was measured between PND21 and 24 in each litter (group PND25 Milk+Solid, n=4 litters) or cage (group PND25 Solid, n=2 cages). C - Body weight measured the day of sampling. The overall effect of experimental groups was tested with a Kruskal-Wallis test. Groups were compared pairwise with Wilcoxon tests. Labeled means without a common letter differ, P<0.05. Barplots show mean values and standard error of the mean.

**Supplementary table 1**: **Chemical composition of the diet**.

|  | Chemical composition (g.kg^-1^) |
| --- | --- |
| Crude protein | 176 |
| Crude fat | 25 |
| Crude cellulose | 114 |
| Acid detergent Lignin | 51 |
| Acid detergent fiber | 165 |
| Neutral detergent fiber | 319 |
| Ash | 64 |
| Starch | 131 |
| Sugars^1^ | 64 |
| Digestible fiber^1^ | 213 |
| Digestible protein^1^ | 125 |
| Digestible energy (MJ.kg^-1^) ^1^ | 9.9 |

^1^ Caculated with WUFFDA software according to tables of ingredients. Maertens L, Pérez JM, Villamide M, Cervera C, Gidenne T, Xiccato G. Nutritive value of raw materials for rabbits: Egran tables 2002. World Rabbit Science 2010;10:157–66.

**Supplementary table 2: Metabolites identified by NMR metabolomics in rabbit caecal content ^1^**.

|  | Metabolite | δ^1^H (ppm) |
| --- | --- | --- |
| 1 | Butyrate | 0.90(t)*, 1.56 (m), 2.16 (t) |
| 2 | Valine | 1.00 (d)*, 1.05 (d), 3.62 (d) |
| 3 | Isoleucine | 0.94 (t)*, 1.01 (d) |
| 4 | Leucine | 0.97 (t)* |
| 5 | Propionate | 1.06 (t), 2.19 (m) |
| 6 | 3-methyl-2-oxovalerate | 1.10 (d)* |
| 7 | 3-methyl-2-oxobutyrate | 1.13 (d)* |
| 8 | Ethanol | 1.19 (t)* |
| 9 | Threonine | 1.34 (d)*, 3.60 (d), 4.26 (m) |
| 10 | Alanine | 1.49 (d)* |
| 11 | Lysine | 1.74 (m)*, 3.03 (t) |
| 12 | Glutarate | 1.79 (m)* |
| 13 | Acetate | 1.92 (s)* |
| 14 | Glutamate | 2.10 (m), 2.35 (m)* |
| 15 | Succinate | 2.41 (s)* |
| 16 | Methylamine | 2.60 (s)* |
| 17 | 4-methyl-2-oxovalerate | 0.94 (d), 2.62 (d)* |
| 18 | Dimethylamine | 2.72 (s)* |
| 19 | Trimethylamine | 2.88 (s)* |
| 20 | 3-phenylpropionate | 2.5 (t), 2.9 (t)*, 7.27 (t), 7.32 (t), 7.38 (t) |
| 21 | Choline | 3.21 (s)* |
| 22 | Glucose | 3.26 (m), 3.38-3.56, 3.7-3.92, 5.24 (d)* |
| 23 | Methanol | 3.36 (s)* |
| 24 | Glycine | 3.57 (s)* |
| 25 | Uracil | 5.80 (d)*, 7.55 (d) |
| 26 | Tyrosine | 6.91 (d)*, 7.20 (d) |
| 27 | Phenylalanine | 7.33 (d), 7.38 (t), 7.43 (t)* |
| 28 | Hypoxanthine | 8.21 (d)* |
| 29 | Formate | 8.46 (s)* |

1: “*” indicates the peak used for quantification based on the corresponding bucket intensity (not overlapping with peaks from other metabolites). Multiplicity of signals is indicated within brackets: s, singlet; d, doublet; t, triplet; m, multiplet.

**Supplementary table 3**: **qPCR primer sequences (5’-3’) targeting rabbit genes**

| Gene name | Forward | Reverse |
| --- | --- | --- |
| *ALPI* | TGCTCTCGGTGATGTACAGG | TCACGTCGATGTCCATGTTG |
| *AQP8* | CCATCGGCTTCTCCGTCGT | CCAATGACGAACCTGATGAGCACT |
| *CA2* | TGGGGCTCATCCGATGGACA | GCCTGGTGTAGCACTGCCAA |
| *CAT* | TCCCGATACTCACCGTCATC | CGTTCAGCACTTTCACGTAGA |
| *CDH1* | GGAACGCCATTGAGGACCCG | GTCATCGTCCGCGTCCGTAG |
| *CLDN1* | TCCAGTGCAAAGTCTTCGAC | CAGCCAGTGAAAAGAGCCTG |
| *CLDN2* | CATCGTGACAGCAGTTGGTT | CCTGAGCAGCCTGGATATCA |
| *CLDN3* | CATGTCTATGGGCCTGGAGA | CTGCACCACACAGTTCATCC |
| *DEFB1* | TGACCATTACAAATGCGCCA | CGTAGCAGGTACCCTCGATT |
| *GAPDH* | AGGTCGGAGTGAACGGATT | ATGGCGACAACATCCACTTT |
| *GPX1* | ATTGAGAATGTGGCGTCGC | CATGAAGTTGGGCTCGAACC |
| *GPX2* | GTAGACTTCAACACGTTCCGG | TTGAGGTAGGTGAAGACGGG |
| *HES1* | CCAAGCTGGAGAAGGCCGATA | GCGGTATTTCCCCAGTACGC |
| *IL18* | TGTAAGCCTCTCTGTGAAGTGT | TCTTATCTTTCTGTCCTGCGAGA |
| *KLF4* | AGAAGCATCTCGGGCAATTC | AGTCGCTTCATGTGGGAGAG |
| *KRT20* | GCTGCAGAACCAGATTAAGGATGC | GCTAGGCGTATCGCCCTCTC |
| *LGR5* | TAACTGGAACTGCGAACCTG | AGCTAAATTCAGGGACCGGA |
| *LYZ* | AGGTGTGAGTTGGCCAGAAC | ACCAGTAGCGGCTATTGATCT |
| *MKI67* | TGGAGTAATCTATGTGGGCCA | CACCAAGAGCCTTTCACCAAA |
| *MUC1* | GCGCTGGCCATCATCTATTT | GCCCTGGTGAATGACATGTC |
| *MUC13* | TTTGGCTACAGTGGAGTGGG | TGGTTTGCGTAGGGGTTTTG |
| *MUC2* | AGCGATGACTTCAGGACGGC | GTCGTGACAGCTGGACTGGG |
| *MYD88* | GGATGGTGGTGGTCGTCTC | TTCTTCATGGCCTTGTACTTGA |
| *MYLK* | CAGAAGGCTGGAGAACGCCG | GGCATAGCTGATGGGCTCGT |
| *NOS2* | GAACTCTCAGCTCATCCGGT | CACCTCGAGCACAATGTCAG |
| *NOX1* | GGTCACTCCCTTTGCTTCCA | TATTGCTGCATGGCCAACAA |
| *NRF2* | AATTCGCCGACTCTGACTCT | TTTTCGAGGACTGGGACTCC |
| *OCLN* | TGGAATGACGGGTCTCTACG | CGGTGTGGACTTGTAGGACT |
| *PCNA* | GCCGAGACCTCAGCCATATT | TGTCCCATGTCGGCAATTTT |
| *PIGR* | GGAGCCATTGACAACCCAAG | CTTGGGCTCCTCAATCTCCA |
| *PYY* | GCTGAACCGCTACTACGCCT | GACCTGACGGGACGGTTCTC |
| *REG3G* | ATGGACATGGATGGGAGTGG | GGGTAACTGCGCATCACAAT |
| *SCL38A3* | ACTGGCCATCACACTACAGA | CAGTGACGGAGACCAGGATT |
| *SLC15A1* | CCCCAAAGCCAATGAAGTCC | TCAAGGCTGGGTGTGATCAT |
| *SLC16A1* | GGCTTCCTTCTGCAATACCG | ACACAGCAGTTCAGTAGGCA |
| *SLC16A10* | TCGGATTCATGTCTGTCCCC | TCTTTTCTCCTCCGGTGCTT |
| *SLC1A5* | TTCAATGGGACCCTGGTGAA | ATGATCCAGGAGACCAGCAC |
| *SLC30A5* | CGGATCCCCTCTGTTCTCTC | CAGAATGGCGCCAGAAATGA |
| *SLC36A1* | GAACCATGCACACTGGGGAA | AAGTTGTCGGCTAGAAACACA |
| *SLC38A2* | CGATGTCAAGCTACCTCTTCA | CAGCAGTGACAGAGGGAGAA |
| *SLC38A5* | CAAAGATGAACGGAGGAGCC | TGGCATTGCTGAGATTGAACA |
| *SLC5A6* | CTGGAGCTGCGGTTCAATA | GTCAGCACTGACAGCCACAG |
| *SLC6A19* | TCCAGACCTGCGACATGAAC | TTCCCGAACATGGACGACAG |
| *SLC7A7* | TGGTACAGGACGTTTTCACCT | GAGCTGAGTACAATGCCAGG |
| *SOD1* | GATTCCATGTCCACCAGTTTG | CCTTTGCCCAAGTCGTCTTC |
| *SOX9* | TGCTGAACGAGAGCGAGAAGC | CTGCCCATTCTTCACCGACTTC |
| *SPDEF* | CCGCTCCATCCGCCAGTATT | CCCTCAGACTGGGTGCACAA |
| *TJP1* | GACCACTCCAGACAGTCTCC | CTGAGTTAGGCAGGACACCA |
| *TJP2* | CAGGGTGGTCATGGTTAACG | GCCTGCTCCTCTCACTGTAT |
| *TLR5* | ACCTCGCCCACAACAAGATA | GGGATCTGAATGTTTGGGCC |
| *VIL1* | CCTCAACTCCAACGATGTCTTC | TGAGTGAAGTCAGGGATCTCC |

**Supplementary table 4: Univariate statistical analysis of bacterial phyla relative abundances in rabbit caecal contents^1^.**

|  | Kruskal-Wallis | Mean relative abundances (%) | | | |
| --- | --- | --- | --- | --- | --- |
| Phylum | p-value | PND18 Milk | PND25 Milk | PND25 Milk+Solid | PND25 Solid |
| Bacteroidota | **0.000** | 60.37 ^a^ | 33.62 ^b^ | 50.35 ^b^ | 47.62 ^b^ |
| Firmicutes | **0.000** | 27.31 ^a^ | 62.76 ^b^ | 47.96 ^b^ | 50.97 ^b^ |
| Actinobacteriota | **0.000** | 0.60 ^ab^ | 1.19 ^a^ | 0.26 ^c^ | 0.51 ^bc^ |
| Campilobacterota | **0.000** | 1.51 ^a^ | 0.02 ^b^ | 0.20 ^c^ | 0.07 ^bc^ |
| Desulfobacterota | **0.047** | 2.12 ^a^ | 0.89 ^a^ | 1.13 ^a^ | 0.59 ^a^ |
| Proteobacteria | **0.003** | 8.09 ^abc^ | 1.52 ^a^ | 0.10 ^b^ | 0.24 ^c^ |

^1^ For each phylum (row), a Kruskal-Wallis test was used. Groups were compared pairwise with a Wilcoxon test. Labeled means without a common letter differ, P<0.05. The mean relative abundance of each phylum in each group is presented. PND: postnatal day. PND18 Milk: n=16, PND25 Milk: n=12, PND25 Milk+Solid: n=12, PND25 Solid: n=12.

**Supplementary table 5: Univariate statistical analysis of bacterial families relative abundances in rabbit caecal contents^1^.**

|  |  | Kruskal-Wallis | | Mean relative abundances (%) | | | |
| --- | --- | --- | --- | --- | --- | --- | --- |
| Phylum | Family | p-value | FDR p-value | PND18 Milk | PND25 Milk | PND25 Milk+Solid | PND25 Solid |
| Actinobacteriota | Atopobiaceae | 0.000 | **0.000** | 0.01 ^a^ | 0.16 ^b^ | 0.08 ^c^ | 0.14 ^bc^ |
| Actinobacteriota | Eggerthellaceae | 0.000 | **0.000** | 0.58 ^ab^ | 1.02 ^a^ | 0.18 ^c^ | 0.37 ^bc^ |
| Bacteroidota | Bacteroidaceae | 0.020 | **0.029** | 30.92 ^ab^ | 19.99 ^a^ | 36.54 ^b^ | 33.71 ^ab^ |
| Bacteroidota | Barnesiellaceae | 0.001 | **0.003** | 8.21 ^a^ | 1.35 ^b^ | 5.12 ^a^ | 4.70 ^a^ |
| Bacteroidota | Marinifilaceae | 0.000 | **0.000** | 1.29 ^a^ | 0.56 ^b^ | 0.51 ^b^ | 0.35 ^b^ |
| Bacteroidota | Muribaculaceae | 0.028 | **0.039** | 0.40 ^a^ | 0.03 ^a^ | 0.76 ^a^ | 0.29 ^a^ |
| Bacteroidota | Prevotellaceae | 0.001 | **0.001** | 0.01 ^a^ | 0.00 ^a^ | 0.53 ^b^ | 0.24 ^b^ |
| Bacteroidota | Rikenellaceae | 0.002 | **0.003** | 17.38 ^a^ | 8.01 ^b^ | 5.18 ^b^ | 7.34 ^b^ |
| Bacteroidota | Tannerellaceae | 0.158 | 0.177 | 2.16 | 3.68 | 1.71 | 0.99 |
| Campilobacterota | Campylobacteraceae | 0.000 | **0.000** | 1.51 ^a^ | 0.02 ^b^ | 0.20 ^c^ | 0.07 ^bc^ |
| Desulfobacterota | Desulfovibrionaceae | 0.047 | 0.062 | 2.12 | 0.89 | 1.13 | 0.59 |
| Firmicutes | [Eubacterium] coprostanoligenes group | 0.000 | **0.001** | 1.25 ^ab^ | 2.29 ^a^ | 0.72 ^b^ | 0.43 ^b^ |
| Firmicutes | Acholeplasmataceae | 0.082 | 0.096 | 0.03 | 0.00 | 0.85 | 0.36 |
| Firmicutes | Anaerofustaceae | 0.000 | **0.000** | 0.02 ^a^ | 0.16 ^b^ | 0.08 ^b^ | 0.11 ^b^ |
| Firmicutes | Anaerovoracaceae | 0.000 | **0.000** | 1.84 ^a^ | 4.89 ^b^ | 0.36 ^c^ | 0.28 ^c^ |
| Firmicutes | Christensenellaceae | 0.000 | **0.000** | 0.22 ^a^ | 1.72 ^b^ | 4.34 ^c^ | 2.79 ^bc^ |
| Firmicutes | Clostridiaceae | 0.275 | 0.285 | 0.01 | 0.00 | 0.27 | 0.12 |
| Firmicutes | Defluviitaleaceae | 0.060 | 0.076 | 0.06 | 0.13 | 0.07 | 0.08 |
| Firmicutes | Lachnospiraceae | 0.015 | **0.024** | 17.23 ^a^ | 28.35 ^ab^ | 20.80 ^ab^ | 23.36 ^b^ |
| Firmicutes | Lactobacillaceae | 0.209 | 0.225 | 0.00 | 0.01 | 0.89 | 0.26 |
| Firmicutes | Monoglobaceae | 0.000 | **0.000** | 0.00 ^a^ | 0.19 ^a^ | 0.75 ^b^ | 1.17 ^b^ |
| Firmicutes | Oscillospiraceae | 0.000 | **0.000** | 5.88 ^a^ | 23.46 ^b^ | 7.36 ^a^ | 6.75 ^a^ |
| Firmicutes | Peptostreptococcaceae | 0.406 | 0.406 | 0.00 | 0.00 | 0.25 | 0.12 |
| Firmicutes | Ruminococcaceae | 0.000 | **0.000** | 0.73 ^a^ | 1.44 ^a^ | 9.10 ^b^ | 12.63 ^b^ |
| Firmicutes | UCG-010 | 0.082 | 0.096 | 0.02 | 0.03 | 0.11 | 0.12 |
| Firmicutes | UCG-011 | 0.001 | **0.002** | 0.00 ^a^ | 0.00 ^a^ | 0.09 ^b^ | 0.20 ^ab^ |
| Proteobacteria | Enterobacteriaceae | 0.000 | **0.000** | 8.05 ^a^ | 1.51 ^a^ | 0.02 ^b^ | 0.04 ^b^ |
| Proteobacteria | Sutterellaceae | 0.000 | **0.000** | 0.04 ^a^ | 0.01 ^a^ | 0.08 ^b^ | 0.19 ^c^ |

^1^ For each family (row), a Kruskal-Wallis test was used and P-values were adjusted with the Benjamini & Hochberg method. Groups were compared pairwise with a Wilcoxon test. Labeled means without a common letter differ, P<0.05. The mean relative abundance of each bacterial family in each group is presented. PND: postnatal day. PND18 Milk: n=16, PND25 Milk: n=12, PND25 Milk+Solid: n=12, PND25 Solid: n=12.

**Supplementary table 6: Univariate statistical analysis of bacterial genera relative abundances in rabbit caecal contents^1^**.

|  |  | Kruskal-Wallis | | Mean relative abundances (%) | | | |
| --- | --- | --- | --- | --- | --- | --- | --- |
| Family | Genus | p-value | FDR  p-value | PND18 Milk | PND25 Milk | PND25 Milk+Solid | PND25 Solid |
| **Actinobacteriota** |  |  |  |  |  |  |  |
| Atopobiaceae | Olsenella | 0.000 | **0.000** | 0.01 ^a^ | 0.16 ^b^ | 0.08 ^c^ | 0.14 ^bc^ |
| Eggerthellaceae | Enterorhabdus | 0.000 | **0.000** | 0.58 ^ab^ | 1.02 ^a^ | 0.18 ^c^ | 0.37 ^bc^ |
| **Bacteroidota** |  |  |  |  |  |  |  |
| Bacteroidaceae | Bacteroides | 0.020 | **0.025** | 30.92 ^ab^ | 19.99 ^a^ | 36.54 ^b^ | 33.71 ^ab^ |
| Marinifilaceae | Butyricimonas | 0.067 | 0.080 | 0.61 | 0.11 | 0.24 | 0.12 |
| Marinifilaceae | Odoribacter | 0.008 | **0.011** | 0.67 ^a^ | 0.45 ^ab^ | 0.27 ^b^ | 0.23 ^b^ |
| Prevotellaceae | Paraprevotella | 0.001 | **0.001** | 0.01 ^a^ | 0.00 ^a^ | 0.53 ^b^ | 0.24 ^b^ |
| Rikenellaceae | Alistipes | 0.015 | **0.021** | 3.25 ^ab^ | 5.05 ^a^ | 1.43 ^ab^ | 1.06 ^b^ |
| Rikenellaceae | dgA-11 gut group | 0.006 | **0.010** | 11.46 ^a^ | 2.96 ^b^ | 2.82 ^b^ | 6.12 ^ab^ |
| Rikenellaceae | Rikenellaceae RC9 gut group | 0.008 | **0.011** | 2.67 ^ab^ | 0.00 ^a^ | 0.94 ^b^ | 0.16 ^ab^ |
| Tannerellaceae | Parabacteroides | 0.158 | 0.183 | 2.16 | 3.68 | 1.71 | 0.99 |
| **Campilobacterota** |  |  |  |  |  |  |  |
| Campylobacteraceae | Campylobacter | 0.000 | **0.000** | 1.51 ^a^ | 0.02 ^b^ | 0.20 ^c^ | 0.07 ^bc^ |
| **Desulfobacterota** |  |  |  |  |  |  |  |
| Desulfovibrionaceae | Desulfovibrio | 0.044 | 0.056 | 1.95 | 0.89 | 0.95 | 0.39 |
| Desulfovibrionaceae | Mailhella | 0.000 | **0.000** | 0.18 ^a^ | 0.00 ^b^ | 0.19 ^a^ | 0.20 ^a^ |
| **Firmicutes** |  |  |  |  |  |  |  |
| Acholeplasmataceae | Anaeroplasma | 0.082 | 0.097 | 0.03 | 0.00 | 0.85 | 0.36 |
| Anaerofustaceae | Anaerofustis | 0.000 | **0.000** | 0.02 ^a^ | 0.16 ^b^ | 0.08 ^b^ | 0.11 ^b^ |
| Anaerovoracaceae | [Eubacterium] nodatum group | 0.000 | **0.001** | 0.29 ^a^ | 0.80 ^a^ | 0.03 ^b^ | 0.03 ^b^ |
| Anaerovoracaceae | Anaerovorax | 0.000 | **0.001** | 0.47 ^a^ | 0.00 ^b^ | 0.01 ^ac^ | 0.00 ^bc^ |
| Anaerovoracaceae | Family XIII AD3011 group | 0.000 | **0.000** | 1.08 ^a^ | 4.09 ^b^ | 0.32 ^ac^ | 0.24 ^c^ |
| Christensenellaceae | Christensenellaceae R-7 group | 0.000 | **0.000** | 0.22 ^a^ | 1.72 ^b^ | 4.34 ^c^ | 2.79 ^bc^ |
| Clostridiaceae | Clostridium sensu stricto 1 | 0.275 | 0.295 | 0.01 | 0.00 | 0.27 | 0.12 |
| Defluviitaleaceae | Defluviitaleaceae UCG-011 | 0.060 | 0.074 | 0.06 | 0.13 | 0.07 | 0.08 |
| Lachnospiraceae | [Eubacterium] hallii group | 0.483 | 0.491 | 0.21 | 0.30 | 0.44 | 0.31 |
| Lachnospiraceae | [Eubacterium] xylanophilum group | 0.000 | **0.000** | 0.00 ^ab^ | 0.00 ^a^ | 0.49 ^bc^ | 0.11 ^c^ |
| Lachnospiraceae | [Ruminococcus] gnavus group | 0.003 | **0.004** | 0.29 ^ab^ | 0.82 ^a^ | 0.11 ^c^ | 0.15 ^bc^ |
| Lachnospiraceae | 28-04 | 0.000 | **0.000** | 0.00 ^a^ | 0.00 ^a^ | 0.25 ^b^ | 0.85 ^b^ |
| Lachnospiraceae | Acetitomaculum | 0.000 | **0.000** | 0.00 ^a^ | 0.06 ^b^ | 0.18 ^b^ | 0.47 ^b^ |
| Lachnospiraceae | Blautia | 0.000 | **0.000** | 0.71 ^a^ | 0.98 ^b^ | 3.87 ^c^ | 4.10 ^c^ |
| Lachnospiraceae | Coprococcus | 0.003 | **0.004** | 0.26 ^a^ | 1.02 ^ab^ | 0.52 ^ab^ | 1.34 ^b^ |
| Lachnospiraceae | Eisenbergiella | 0.000 | **0.000** | 0.01 ^a^ | 0.02 ^ab^ | 0.57 ^bc^ | 0.26 ^c^ |
| Lachnospiraceae | Frisingicoccus | 0.002 | **0.003** | 0.08 ^a^ | 0.37 ^b^ | 0.20 ^ab^ | 0.08 ^a^ |
| Lachnospiraceae | Fusicatenibacter | 0.000 | **0.000** | 8.72 ^a^ | 4.27 ^ab^ | 2.17 ^b^ | 2.34 ^b^ |
| Lachnospiraceae | GCA-900066575 | 0.000 | **0.000** | 0.00 ^a^ | 0.00 ^a^ | 0.28 ^b^ | 0.59 ^b^ |
| Lachnospiraceae | Hespellia | 0.000 | **0.000** | 0.00 ^a^ | 0.00 ^a^ | 0.32 ^b^ | 0.09 ^b^ |
| Lachnospiraceae | Hungatella | 0.558 | 0.558 | 0.00 | 0.01 | 0.26 | 0.02 |
| Lachnospiraceae | Lachnoclostridium | 0.018 | **0.024** | 0.17 ^a^ | 3.73 ^b^ | 0.90 ^ab^ | 1.98 ^ab^ |
| Lachnospiraceae | Lachnospiraceae NK4A136 group | 0.018 | **0.024** | 0.29 ^a^ | 0.18 ^a^ | 3.01 ^b^ | 1.15 ^ab^ |
| Lachnospiraceae | Lachnospiraceae NK4B4 group | 0.000 | **0.000** | 0.00 ^a^ | 0.00 ^a^ | 0.31 ^b^ | 0.34 ^b^ |
| Lachnospiraceae | Lachnospiraceae UCG-006 | 0.000 | **0.000** | 0.52 ^a^ | 7.15 ^b^ | 0.60 ^a^ | 0.28 ^a^ |
| Lachnospiraceae | Marvinbryantia | 0.000 | **0.000** | 0.75 ^a^ | 2.80 ^b^ | 2.03 ^b^ | 4.74 ^b^ |
| Lachnospiraceae | Roseburia | 0.009 | **0.013** | 0.93 ^a^ | 0.07 ^b^ | 0.10 ^b^ | 0.35 ^ab^ |
| Lachnospiraceae | Tyzzerella | 0.000 | **0.000** | 0.00 ^a^ | 0.00 | 0.62 ^b^ | 1.33 ^b^ |
| Lactobacillaceae | Lactobacillus | 0.209 | 0.233 | 0.00 | 0.01 | 0.89 | 0.26 |
| Monoglobaceae | Monoglobus | 0.000 | **0.000** | 0.00 ^a^ | 0.19 ^a^ | 0.75 ^b^ | 1.17 ^b^ |
| Oscillospiraceae | Colidextribacter | 0.000 | **0.000** | 0.67 ^a^ | 0.00 ^b^ | 0.62 ^a^ | 0.86 ^a^ |
| Oscillospiraceae | NK4A214 group | 0.000 | **0.000** | 4.09 ^a^ | 21.41 ^b^ | 4.88 ^a^ | 4.87 ^a^ |
| Oscillospiraceae | Oscillibacter | 0.229 | 0.251 | 0.15 | 0.09 | 0.09 | 0.09 |
| Oscillospiraceae | Oscillospira | 0.000 | **0.000** | 0.14 ^a^ | 0.49 ^b^ | 0.05 ^a^ | 0.06 ^a^ |
| Oscillospiraceae | UCG-003 | 0.000 | **0.000** | 0.02 ^a^ | 0.01 ^b^ | 0.11 ^c^ | 0.30 ^c^ |
| Oscillospiraceae | V9D2013 group | 0.334 | 0.352 | 0.14 | 0.15 | 0.48 | 0.09 |
| Peptostreptococcaceae | Terrisporobacter | 0.406 | 0.420 | 0.00 | 0.00 | 0.25 | 0.12 |
| Ruminococcaceae | [Eubacterium] siraeum group | 0.000 | **0.001** | 0.00 ^a^ | 0.00 ^a^ | 0.17 ^b^ | 0.37 ^b^ |
| Ruminococcaceae | CAG-352 | 0.000 | **0.000** | 0.00 ^a^ | 0.00 ^a^ | 1.11 ^b^ | 1.37 ^b^ |
| Ruminococcaceae | Harryflintia | 0.206 | 0.233 | 0.26 | 0.22 | 0.03 | 0.03 |
| Ruminococcaceae | Paludicola | 0.000 | **0.000** | 0.00 ^a^ | 0.00 ^a^ | 0.84 ^b^ | 0.60 ^b^ |
| Ruminococcaceae | Ruminococcus | 0.000 | **0.000** | 0.02 ^a^ | 0.00 ^b^ | 4.93 ^c^ | 5.77 ^c^ |
| Ruminococcaceae | UBA1819 | 0.000 | **0.000** | 0.16 ^a^ | 0.73 ^b^ | 0.07 ^a^ | 0.06 ^a^ |
| **Proteobacteria** |  |  |  |  |  |  |  |
| Enterobacteriaceae | Escherichia-Shigella | 0.000 | **0.000** | 8.05 ^a^ | 1.51 ^a^ | 0.02 ^b^ | 0.04 ^b^ |
| Sutterellaceae | Parasutterella | 0.000 | **0.000** | 0.04 ^a^ | 0.01 ^a^ | 0.08 ^b^ | 0.19 ^c^ |

^1^ For each genus (row), a Kruskal-Wallis test was used and P-values were adjusted with the Benjamini & Hochberg method. Groups were compared pairwise with a Wilcoxon test. Labeled means without a common letter differ, P<0.05. The mean relative abundance of each bacterial genus in each group is presented. PND: postnatal day. PND18 Milk: n=16, PND25 Milk: n=12, PND25 Milk+Solid: n=12, PND25 Solid: n=12.

**Supplementary table 7**: **Univariate statistical analysis of metabolites relative concentrations in rabbit caecal contents** **^1^**.

|  | Kruskal-Wallis | | Mean relative concentrations | | | |
| --- | --- | --- | --- | --- | --- | --- |
| Metabolite | p-value | FDR  p-value | PND18 Milk | PND25 Milk | PND25 Milk+Solid | PND25  Solid |
| 3-methyl-2-oxobutyrate | 0.043 | **0.043** | 0.0004^a^ | 0.0002^b^ | 0.0005^ab^ | 0.0003^ab^ |
| 3-methyl-2-oxovalerate | 0.006 | **0.008** | 0.001^a^ | 0.0007^ab^ | 0.0004^b^ | 0.0005^b^ |
| 3-phenylpropionate | 0.000 | **0.000** | 0.0006^a^ | 0.0003^b^ | 0.0013^c^ | 0.0024^d^ |
| 4-methyl-2-oxovalerate | 0.000 | **0.000** | 0.0008^a^ | 0.0004^b^ | 0.0002^c^ | 0.0001^d^ |
| Acetate | 0.000 | **0.000** | 0.0908^a^ | 0.1666^b^ | 0.3396^c^ | 0.3761^c^ |
| Alanine | 0.000 | **0.000** | 0.0067^a^ | 0.0057^a^ | 0.0021^b^ | 0.0019^b^ |
| Butyrate | 0.000 | **0.000** | 0.0035^a^ | 0.0094^b^ | 0.0435^c^ | 0.0352^c^ |
| Choline | 0.000 | **0.000** | 0.0075^a^ | 0.0092^a^ | 0.0011^b^ | 0.001^b^ |
| Dimethylamine | 0.000 | **0.000** | 0.0011^a^ | 0.0004^b^ | 0.0009^a^ | 0.0004^b^ |
| Ethanol | 0.038 | **0.039** | 0.0035^a^ | 0.009^a^ | 0.0053^a^ | 0.004^a^ |
| Formate | 0.000 | **0.000** | 0.0009^a^ | 0.0008^a^ | 0.0006^b^ | 0.0005^b^ |
| Glucose | 0.000 | **0.000** | 0.0007^a^ | 0.0013^b^ | 0.0038^c^ | 0.0051^c^ |
| Glutamate | 0.000 | **0.000** | 0.0046^a^ | 0.0078^b^ | 0.0042^ac^ | 0.003^c^ |
| Glutarate | 0.030 | **0.032** | 0.0009^a^ | 0.0011^ab^ | 0.0012^ab^ | 0.0016^b^ |
| Glycine | 0.000 | **0.000** | 0.0057^a^ | 0.0057^a^ | 0.0021^b^ | 0.0021^b^ |
| Hypoxanthine | 0.000 | **0.000** | 0.0007^a^ | 0.0005^b^ | 0.0009^a^ | 0.0003^c^ |
| Isoleucine | 0.000 | **0.000** | 0.0029^a^ | 0.0036^a^ | 0.0006^b^ | 0.001^b^ |
| Leucine | 0.000 | **0.000** | 0.0082^a^ | 0.0094^a^ | 0.0012^b^ | 0.0014^b^ |
| Lysine | 0.000 | **0.000** | 0.0031^a^ | 0.0036^a^ | 0.0011^b^ | 0.0009^b^ |
| Methanol | 0.028 | **0.031** | 0.014^a^ | 0.0171^a^ | 0.0178^a^ | 0.0183^a^ |
| Methylamine | 0.001 | **0.001** | 0.0022^a^ | 0.0019^a^ | 0.0018^a^ | 0.0009^b^ |
| Phenylalanine | 0.000 | **0.000** | 0.0016^a^ | 0.0013^a^ | 0.0003^b^ | 0.0003^b^ |
| Propionate | 0.000 | **0.000** | 0.0052^a^ | 0.0209^b^ | 0.0169^c^ | 0.0123^d^ |
| Succinate | 0.000 | **0.000** | 0.0052^a^ | 0.0093^a^ | 0.0009^b^ | 0.0013^b^ |
| Threonine | 0.000 | **0.000** | 0.003^ab^ | 0.0036^a^ | 0.0018^b^ | 0.0016^b^ |
| Trimethylamine | 0.000 | **0.000** | 0.0138^a^ | 0.0055^b^ | 0.0039^b^ | 0.0037^b^ |
| Tyrosine | 0.000 | **0.000** | 0.0019^a^ | 0.0015^a^ | 0.0003^b^ | 0.0003^b^ |
| Uracil | 0.000 | **0.000** | 0.0012^a^ | 0.0008^b^ | 0.0003^c^ | 0.0002^d^ |
| Valine | 0.000 | **0.000** | 0.0033^a^ | 0.0028^a^ | 0.0004^b^ | 0.0003^b^ |

^1^ For each metabolite (row), a Kruskal-Wallis test was used and P-values were adjusted with the Benjamini & Hochberg method. Groups were compared pairwise with a Wilcoxon test. Labeled means without a common letter differ, P<0.05. The mean relative concentration of each metabolite in each group is presented. PND: postnatal day. PND18 Milk: n=16, PND25 Milk: n=12, PND25 Milk+Solid: n=12, PND25 Solid: n=12.
